# Temperature-Dependent Voltammetry Reveals the Potential-Dependent Formation of Reaction Species and Their Impact on Interfacial Excess Charge during the Ammonia Oxidation Reaction on Pt

**DOI:** 10.1021/jacs.6c04573

**Published:** 2026-09-03

**Authors:** Francisco Sarabia, Sojung Park, Beatriz Roldan Cuenya, Sebastian Z. Oener

**Affiliations:** Department of Interface Science, 28259 Fritz-Haber Institute of the Max Planck Society, Berlin 14195, Germany

## Abstract

The electrocatalytic ammonia oxidation reaction (AOR) has historically garnered significant interest, since ammonia could be used as a carbon-free fuel and convenient H_2_ carrier. Key challenges hindering its application are the high overpotentials and rapid catalyst poisoning, which remain poorly understood. Here, we study the overpotential-dependent formation of reaction species via temperature-dependent cyclic voltammetry and electrochemical Arrhenius analysis on polycrystalline Pt in conjunction with infrared spectroscopy. Further, we link the evolution of the kinetic parameters to the degree of polarization and excess charge via CO-displacement measurements. Strikingly, with increasing overpotential, we discover the parallel formation of a reaction species (not a direct intermediate) that modulates the electron chemical potential and, thus, the potential of zero charge. Due to this coupling, the parallel formation of this “poisoning” species appears to depolarize the surface with increasing overpotential. To recover the interfacial polarization, the overpotential needs to be increased further, before the activity can be restored and substantial Faradiac current can arise. What emerges is a new kinetic picture, where not only formation but also stabilization of interfacial excess charge are decisive for the catalyst activity. In general, the study demonstrates the utility of performing temperature-dependent cyclic voltammetry for revealing how transient changes in local chemicals modulate the overall inner-sphere kinetics.

## Graphical abstract

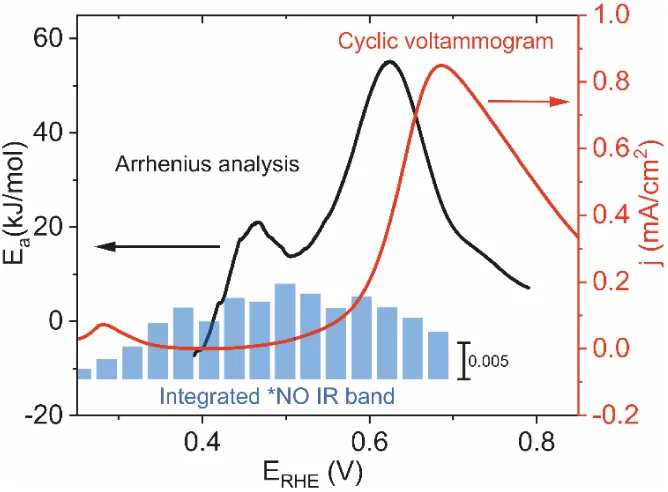

## Introduction

Gaining mechanistic insight into the electrochemical ammonia oxidation reaction (AOR) is essential to utilizing green ammonia both as a hydrogen carrier and as a fuel for low-temperature electrochemical technologies.^1–3^ For example, H_2_ can be extracted from NH_3_ by electrocatalytic oxidation to N_2_ and formation of water in alkali that is then reduced to H_2_ at the cathode. The cell potential of the balanced reaction is only ∼50 mV at standard state, i.e., the NH_3_ essentially provides a chemical bias to the cell, compared to a cell potential of 1.23 V when starting from H_2_O at standard state. However, the reaction is challenged by multiple factors. Extensive electrochemistry,^4–6^ spectroscopy,^7–9^ and density functional theory^10–13^ studies established that expensive Pt- and Ir-based catalysts are the most active AOR catalysts in aqueous media. Further, even for the Pt-group metals, large overpotentials and rapid catalyst deactivation are observed that are poorly understood due to the complicated multistep reaction sequence.^14–17^

The Gerischer–Maurer mechanism serves as the foundation for electrocatalytic ammonia oxidation, with modifications proposed by the groups of Janik, Koper, and others (Figure 1).^10,18,19^ In this context, the overall reaction (6OH^–^ + 2NH_3_ → 6e^–^+ N_2_ + 3H_2_O) can be described by a succession of presteps, before two parallel pathways emerge, one leading to the desired N_2_ and the other to a poisoning species, likely N_(2)_O according to spectroscopic evidence.^9,10,20^ To form the desired N_2_, not only six electrons need to be stripped from two NH_3_ molecules, but also several N_*x*_H_*y*_-intermediates need to be deprotonated via OH^–^ attack from solution. In fact, Katsounaros et al. proposed a hydroxide-decoupled electron transfer step (*k*_2_ in Figure 1) that leads to a negatively charged intermediate (simplified as NH^–^ in Figure 1), to explain the pH-dependent AOR activity.^4,10^ Based on DFT, this intermediate was found to have a suitable binding energy and it was argued to be the rate-determining intermediate, i.e., the complicated multistep sequence was essentially reduced to the energetics of one intermediate.

**Figure 1:**
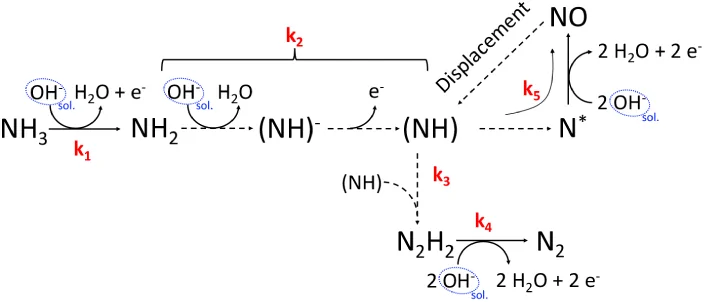
Different pathways for NH_3_ oxidation on Pt. The schematic shows several distinct elementary steps with different rate constants (*k*_1_–*k*_5_). In our work, overpotential-dependent rate-limiting steps (r_ls_) are considered, involving the transformation of adsorbed NH_3_ into NH_2_, NH^–^ (simplified from (N*_x_* H*_y_*)^−^), N_2_H_2_, and N_2_. A poisonous species, such as NO, likely displaces the intermediates on a ms-timescale and modulates the potential of zero charge. As detailed in the text, the availability of solvated (sol.) hydroxides (dotted circles) might be critical for this complicated reaction sequence. Whereas the schematic depicts key aspects of the AOR based on previous work,^4,10,11,18^ the role of the desolvation and transfer of interfacial hydroxide ions was not captured in previous theory and most experiments.

Recently, we performed extensive overpotential-dependent Arrhenius analyses, including analysis with millisecond time resolution to study the AOR over a surface that is transiently poisoned.^21^ We observed a compensation effect at low overpotentials, which is very prevalent across reactions and catalysts,^21–26^ where increasing rates coincide with an increasing apparent activation energy (*E*_a_) that is overcompensated by an increasing pre-exponential factor (log *A*). We proposed that under certain conditions, the increasing *E*_a_ is related to increasing excess charge and electric fields across the water dielectric that are needed to desolvate hydroxide ions from the bulk (Figure 1).^21–23^ More specifically, enough polarization must develop across the electrode–solution interface to induce a solvent structure that facilitates proton and hydroxide transfer. The extent of this interfacial polarization can be modulated partly with the applied potential and species that form during the reaction. Within this framework, a pH-dependent formation of excess charge could also explain pH-dependent AOR activity. In fact, with decreasing pH, we observed not only decreasing AOR activity but also a decreasing apparent activation energy. Consequently, under less alkaline conditions, a higher overpotential is required to achieve a pre-exponential factor comparable to that attained under strongly alkaline conditions. More recently, molecular dynamics and grand canonical DFT calculations by Parrinello and coworkers for the CO_2_ reduction reaction^27^ have shown that proton and hydroxide transfer in the electric-field-dependent hydrogen-bonded network can greatly dominate the free-energy changes of an electrocatalytic reaction. This supports our hypothesis on the importance of ion-solvation (pre)­steps.^21–23^

In general, for multistep reactions, the apparent activation parameters cannot be reduced to the impact of one rate-determining step or transition state with a constant degree of rate control.^28–32^ In fact, when we extended the current and pressure ranges of Arrhenius analysis in a fuel cell environment, we discovered very rich overpotential-dependent oscillations of the apparent activation parameters for the multistep oxygen reduction reaction (ORR)^25^ that we linked to structural and chemical changes at the metal-solution interface,^25^ and further explored the multistep nature with a simple microkinetic model.^30^ However, in contrast to the ORR, high current density operation of the AOR is plagued by rapid catalyst deactivation.^21^ Therefore, the question arises whether the overpotential-dependent formation of intermediates in multistep reactions can be revealed even before the onset of substantial Faradaic current, and how such intermediates are linked to the overpotential-dependent activation parameters. In general, for multistep reactions that proceed at high overpotentials, there is no reason to expect that important reaction species or intermediates only form once Faradaic current arises. In fact, such an assumption that can often be found in the literature likely obscures the fundamental origin of the kinetic overpotential for multistep reactions.

Here, we study the overpotential-dependent formation of key intermediates during the AOR multistep sequence and reveal that the formation of a parallel species modulates the electronic polarization. What emerges is a new kinetic picture where the overpotential-dependent formation of intermediates is closely tied to the stabilization of interfacial excess charge, which might be decisive for understanding the very origin of the overpotential across many electrocatalytic reactions.

### Electrochemical Arrhenius Analysis of Temperature-Dependent Cyclic Voltammetry

To inform on the total charge and coverage of different intermediates, we stepped the potential from an anodic potential region (where the adsorbed intermediates form) toward a reductive stripping potential and analyzed the transient stripping current. The potential of zero total charge (pztc) was determined via CO charge displacement as a function of NH_3_ concentration. Arrhenius analysis was based on temperature-dependent cyclic voltammetry (10–30 °C in 5 °C increments) at various scan rates (10, 20, 50, 100, and 200 mV s^–1^) and NH_3_ concentrations (10^–3^, 10^–2^, and 10^–1^ M) with regression R^2^ values above 0.95 (Supplementary Figures 1–6 and Supplementary Note 1).

Figure 2A shows the voltammetric profile for NH_3_ oxidation on polycrystalline Pt at 25 °C in alkaline medium at different NH_3_ concentrations. The current observed above 0.55 V_RHE_ (potential referenced vs the reversible hydrogen electrode, RHE) corresponds to NH_3_ oxidation toward N_2_ formation, along with parallel pathways that generate poisoning species leading to catalyst deactivation.^5,33^ Below 0.55 V_RHE_, however, not only NH_3_ adsorption occurs, but also the first oxidation step, as suggested in previous studies.^7,9^ These processes are reflected in the charge distribution observed in the cyclic voltammograms at different ammonia concentrations. At the lowest ammonia concentration, two peaks are observed around 0.25 V_RHE_ and 0.33 V_RHE_, which are likely associated with OH^–^/H^+^ adsorption–desorption processes on the (110) and (100) atomic orientations of the polycrystalline Pt surface, respectively.^34,35,36^ As the ammonia concentration increases, the first peak disappears, whereas the second one shifts toward lower potentials (for more details, see Supplementary Figure 7). The distinct evolution of these peaks may be attributed to differences in the interaction of nitrogen-containing species with the two facets, arising from their specific surface atomic arrangements, intermediate chemical potentials, surface charge densities, solvent interactions, and other interfacial factors, such as NH_3_-dependent restructuring. The suppression of the peak associated with the Pt(110) facet indicates a strong interaction between this surface and nitrogen-containing species. As reported in the literature, this strong interaction leads to negligible catalytic activity. In contrast, the Pt(100) surface allows more reversible adsorption of nitrogen-containing species and exhibits the highest catalytic activity for AOR.

**Figure 2:**
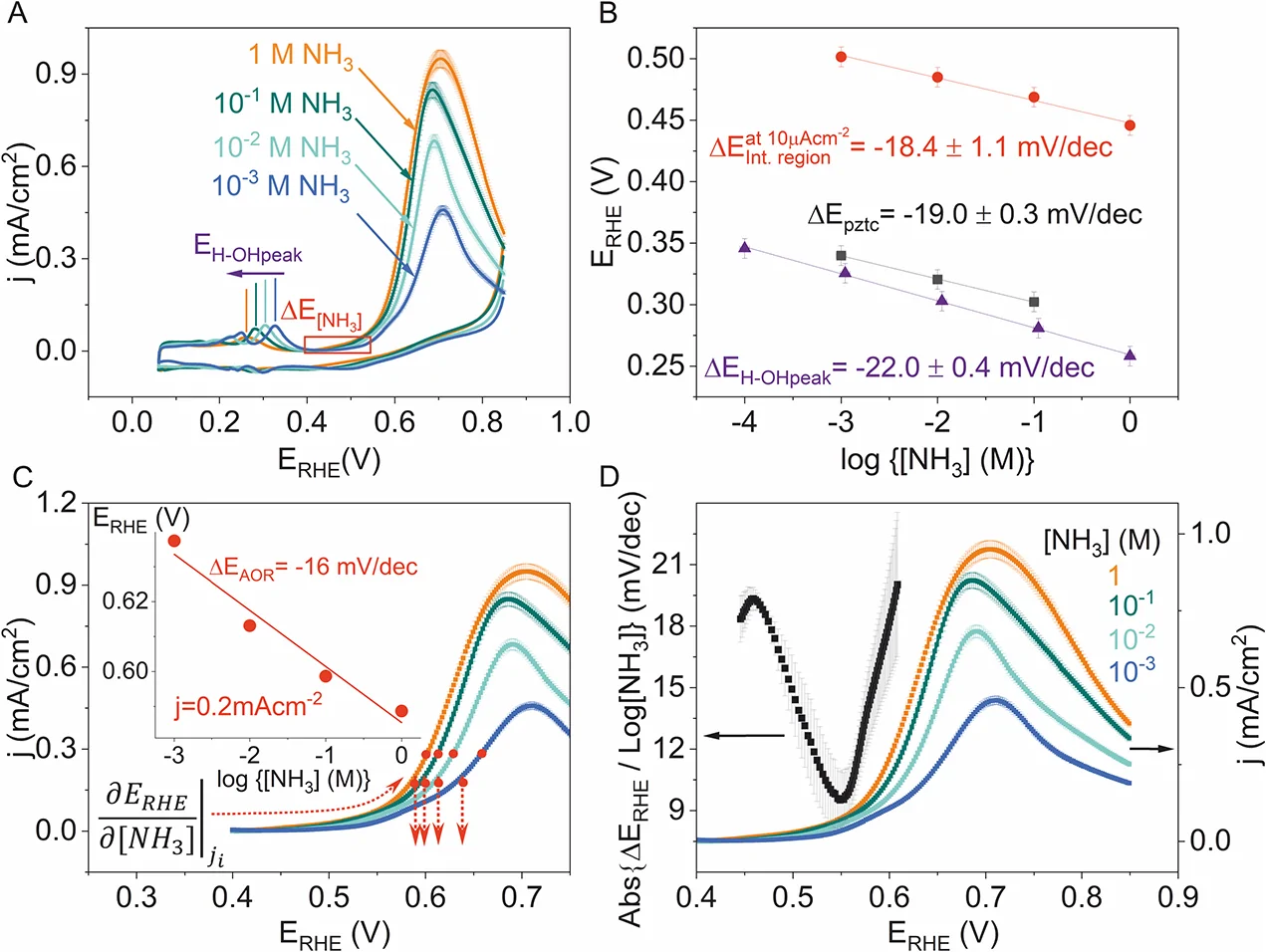
Impact of NH_3_ concentration on the H^+^/OH^–^ adsorption–desorption region (*E* < 0.4 V_RHE_), an intermediate region (0.4 V_RHE_ < *E* < 0.55 V_RHE_) and during N_2_ evolution (*E* > 0.55 V_RHE_). (**A**) Cyclic voltammograms of polycrystalline Pt at different NH_3_ concentrations, [NH_3_]. (**B**) *E* at 10 μA cm^–2^, taken as onset potential for the AOR in the intermediate region between the H^+^/OH^–^ adsorption region and full ammonia oxidation (red circles), pztc (black squares), and second H^+^/OH^–^ peak (purple triangles) vs log­[NH_3_]. (**C**) Procedure to extract *E* vs log­[NH_3_] at fixed current. (**D**) *E* variation with [NH_3_] referenced to 1 M (left axis) and AOR current (right axis). For all measurements, 0.1 M KOH at 25 °C and a scan rate of 50 mV s^–1^ were used.

Charge analysis of the H^+^/OH^–^ adsorption region indicates at least a first deprotonation of NH_3_, as the total charge involved in the cyclic voltammograms varies systematically with the NH_3_ concentration (see Supplementary Note 2). Importantly, increasing NH_3_ concentration does not lead to a constant or progressively decreasing charge, which would be expected from simple surface blockage. Instead, the charge first decreases relative to the blank due to initial site blocking, and subsequently increases with further NH_3_ addition, consistent with the formation of early intermediates. At even higher concentrations, the charge decreases again, likely reflecting a change in the stoichiometry between nitrogen-containing species and Pt surface atoms. Notably, the presence of NH_2_ adsorbates within this potential region (see Supplementary Figures 8–9) has been reported in previous infrared spectroscopy studies.^9^ Therefore, this shift observed in the prepeak associated with OH^–^/H^+^ adsorption suggests a change in the surface chemical potential driven by the evolving coverage of NH_3_ and NH_3–x_ species, where x denotes the number of protons removed upon partial deprotonation at <0.4 V_RHE_. As shown in Figure 2B, a strong correlation between the electron chemical potential and the coverage of N-containing species is further supported by the comparable dependence of both the potential of zero total charge (pztc) and the prepeak potential on the NH_3_ concentration, with slopes of 19 and 22 mV dec^–1^, respectively. For details about the determination of the pztc, see Supplementary Note 3.

The dependence of the pztc on the intermediate coverage reflects concentration-dependent variations in the electron chemical potential. Therefore, we hypothesized that the observed shift in the cyclic voltammogram onset >0.4 V_RHE_ with increasing NH_3_ concentration is likewise impacted by the underlying trend in chemical potential variation. Figure 2C outlines a protocol to extract the dependence of the potential, *E*, at a fixed current density, j_i_, on log­[NH_3_]. This way, we can evaluate how a changing concentration of NH_3_, or the associated intermediates, impacts either pseudocapacitive adsorption or Faradaic kinetics. In Figure 2D, we show the resulting slope, Δ*E*/log­[NH_3_], as a function of potential and referenced to the cyclic voltammogram at 1 M NH_3_. The right *y*-axis in Figure 2D shows the corresponding currents from the cyclic voltammogram with a scan rate of 50 mV s^–1^ at different NH_3_ concentrations. Strikingly, Δ*E*/log­[NH_3_] initially exhibits a slope close to 20 mV dec^–1^, matching the dependence on the NH_3_ concentration of both the pztc and the prepeak (Figure 2B). With increasing potential, the slope falls to ∼9 mV dec^–1^ before it increases again. This hints toward the formation of a parallel species to N_2_ evolution <0.55 V_RHE_ (referenced to 1 M NH_3_). Based on the IR spectroscopic results presented later, we assign this species to adsorbed *NO.

To test for the formation of a reaction product between 0.4 and 0.55 V_RHE_, we performed chronoamperometric charge analysis. In this approach, adsorbed species are formed during a controlled interval at >0.4 V_RHE_ and subsequently reductively stripped by stepping the potential to 0.1 V_RHE_ (see Supplementary Note 4 for details). Importantly, this potential window overlaps with the double-layer potential window of Pt, which is free of substantial structural and oxidation changes. Figure 3A shows the (sign-inverted) reductive desorption current of these species vs potential (left *y*-axis). The current increases and reaches a saturation value at potentials of ∼0.52 V_RHE_. Importantly, the saturation does not correspond to the maximum coverage of a full monolayer, as the current continues to increase with the NH_3_ concentration.

**Figure 3:**
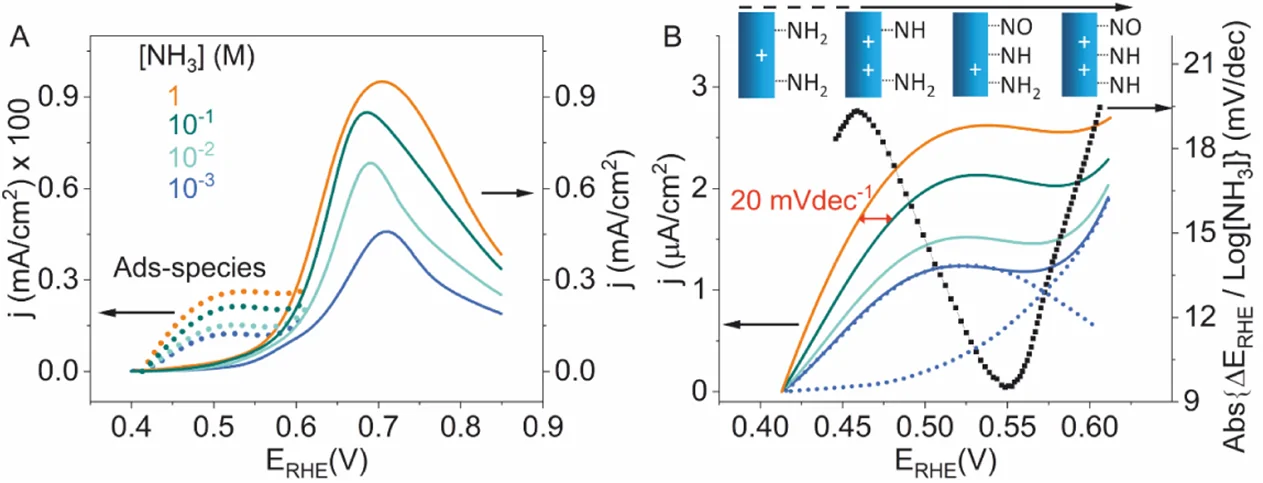
Coulometric evidence of reaction intermediates. **(A)** Current vs potential associated with the adsorbed species (left axis, extracted from potential-jump studies; see Supplementary Note 4) and NH_3_ oxidation current (right axis). (**B**) Comparison of the *NO species-related current (left axis) with Δ*E* (right axis). The dotted blue lines illustrate the contribution of different intermediates to the total current of adsorbed species at 1 mM NH_3_. The inset schematically depicts the variation of the excess charge associated with the overpotential-dependent formation of *NO, which affects the subsequent formation of NH from NH_2_ species. For all measurements, 0.1 M KOH at 25 °C and a scan rate of 50 mV s^–1^ were used. For panel B, the potential was stepped and held (30 s) at 0.413 V_RHE_, 0.463 V_RHE_, 0.513 V_RHE_, and 0.613 V_RHE_. Afterward, the potential was stepped and held (4 s) at 0.1 V_RHE_ to strip the intermediates. The current associated with the adsorbed species was calculated by dividing the charge measured during the potential-jump experiments by the corresponding charge-recording time (see Supplementary Note 4).

Figure 3B shows that the behavior of the reductive desorption currents implies an important role in the behavior of Δ*E*/log­[NH_3_] for 0.4–0.55 V_RHE_. Initially, for a low fixed current density, e.g., 0.25 μA cm^–2^, increasing the NH_3_ concentration leads to a shift of the respective (over)­potential. At 1.7 μA cm^–2^, the shift in potential between 0.1 and 1 M NH_3_ (orange and green lines) matches the Δ*E*/log­[NH_3_] of 20 mV dec^–1^. At higher potentials, the current associated with the adsorbed species saturates, which dominates the current–potential response and, thus, does not show a simple (over)­potential shift with NH_3_ concentration. A saturation behavior can qualitatively explain the substantial reduction of Δ*E*/log­[NH_3_] to ∼9 mV dec^–1^.

Above 0.55 V_RHE_, Δ*E*/log­[NH_3_] rises again, recovering to and potentially exceeding the initial values. Importantly, the current saturates as a function of overpotential up to 0.57 V_RHE_, but increases with NH_3_ concentration. The dotted lines tentatively indicate the current associated with the parallel formation of adsorbed species and the onset current of the Faradaic AOR in Figure 3B for 10^–3^ M NH_3_ (blue line). Collectively, these results strongly suggest that at 0.4–0.55 V_RHE_ a parallel adsorbed species is formed that is not part of the primary AOR sequence. This species impacts the AOR mechanism and displays potential shifts that initially closely match the ones of the pztc and AOR overpotentials with NH_3_ concentration (Figure 2B). However, as shown in Figure 3B, Δ*E* substantially decreases with increasing coverage in the saturation region.

The formation of an *NO species could induce changes in the electrode–solvent polarization, as schematically depicted in the inset in Figure 3B and discussed later. At 0.4 V_RHE_, Δ*E*/log­[NH_3_] shows the same shift as the pztc with NH_3_ concentration (Figure 2B), strongly suggesting an important link between the excess charge and the formation of the parallel species. However, with increasing coverage of the *NO species, Δ*E*/log­[NH_3_] is reduced and the *NO current saturates. We hypothesize that the increasing coverage of *NO might partially depolarize the surface between 0.4 and 0.55 V_RHE_. Note that the pztc shift with NH_3_ concentration in Figure 2B cannot account for this, as the measurements were performed in the H underpotential region in the presence of NH_3_ and the first intermediate NH_2_.

Our results strongly suggest that the Δ*E*/log­[NH_3_] and pztc/log­[NH_3_] are governed by the potential-dependent formation of species at the electrode surface. Based on the spectroscopic results presented below, we attribute the change in the surface chemical potential to the formation of *NO. This is also consistent with our previous results on the AOR, where we performed time-dependent electrochemical Arrhenius analysis to study the AOR with millisecond time resolution over a surface that is dynamically covered by a poisoning species.^21^ Thus, we explored temperature-dependent cyclic voltammograms as a means to reveal the formation of species in the multistep AOR sequence, not only during, but also before the onset of the Faradaic reaction.

Figure 4A–B present the potential-dependent *E_a_* and log *A* obtained from Arrhenius analysis of cyclic voltammetry at different scan rates (see Supplementary Note 1 for details). For a given scan rate, both kinetic parameters reveal two clear maxima, one at 0.45 V_RHE_ and one at 0.6–0.65 V_RHE_, separated by a minimum between ∼0.45–0.55 V_RHE_. To establish a closer relation between the apparent activation parameters and the overpotential-dependent formation of intermediates, Figure 4C compares the apparent *E*_a_ for 0.1 M NH_3_ at 50 mV s^–1^ (as a representative kinetic descriptor) with Δ*E*/log­[NH_3_] and the total NH_3_ oxidation current, and the parallel species current from Figure 3. Starting at 0.4 V_RHE_, the apparent *E*_a_ and log *A* increase together, paralleled by an increase in Δ*E*/log­[NH_3_]. After this maximum, *E*_a_ and log *A* decrease despite the fact that the intermediate coverage saturates between 0.45 and 0.55 V_RHE_. However, in this region Δ*E*/log­[NH_3_] decreases too.

**Figure 4:**
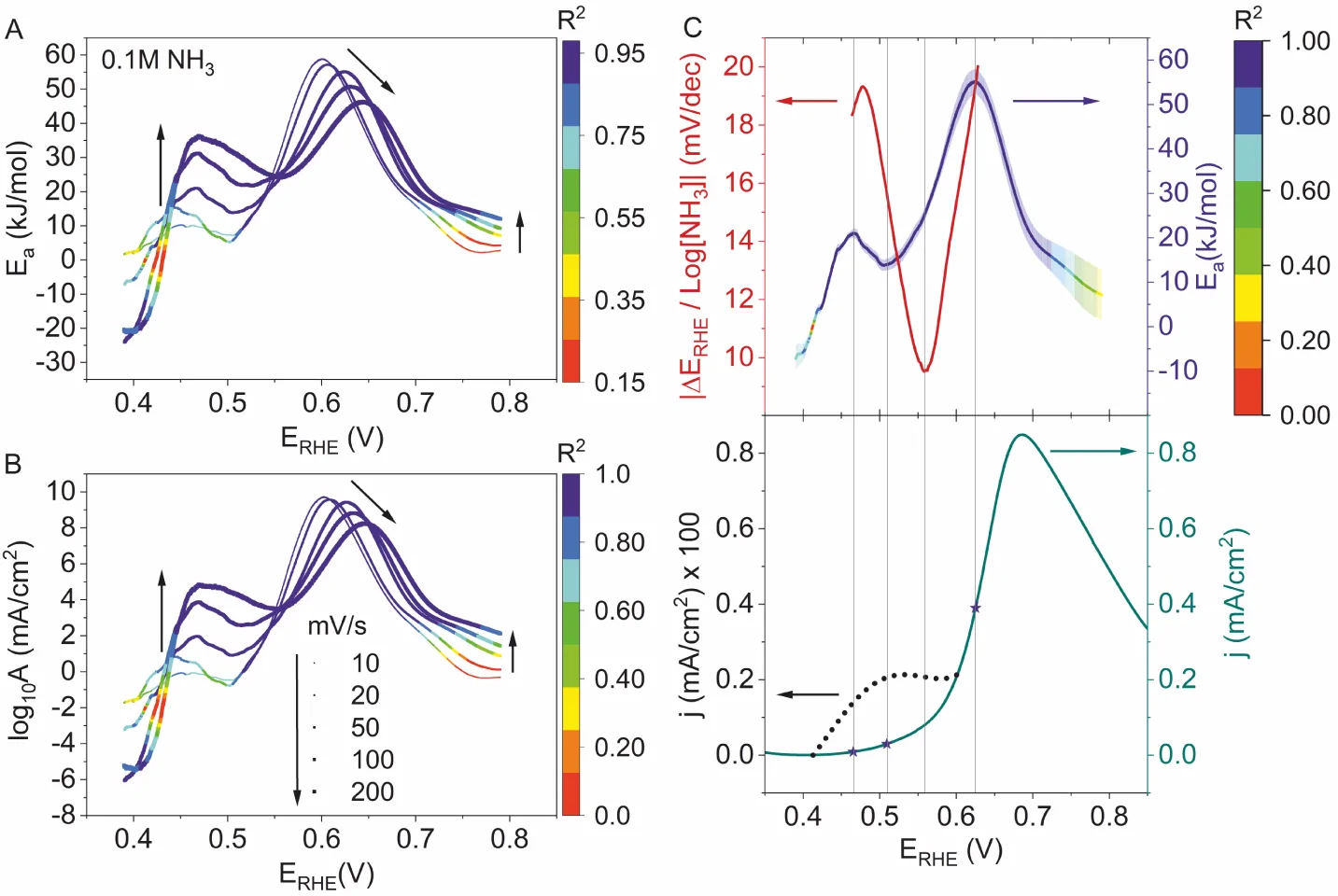
Evolution of kinetic parameters with potential and their correlation with the formation of adsorbed species. (**A**) Potential-dependent activation energy (*E*_a_) and (**B**) logarithm of the pre-exponential factor (log *A*) extracted from voltammograms at different temperatures (see Supplementary Note 1) and scan rates. (**C**) Correlation of *E*_a_ (top, right axis) and Δ*E*_RHE_/log­[NH_3_] (top, left axis) with the variation in adsorbed-species-related current (bottom, left axis, black dotted line) and total oxidation current at 0.1 M NH_3_ (bottom, right axis, green solid line) vs *E*_RHE_. Kinetic parameters are shown with a color code reflecting the R^2^ value from the linear regression at each point, based on five temperatures (10–30 °C). For all experiments, 0.1 M KOH was used.

The close correlation between the apparent activation parameters and Δ*E*/log­[NH_3_], which matches pztc/log­[NH_3_] in various regimes (Figure 2B), and the fact that *E*_a_ and log *A* decrease again despite a saturating intermediate species, support our hypothesis that increasing *E*_a_ and log *A* may be strongly dominated by increasing excess charge and electric fields inside the double layer.^21^ This will be further supported below when we study the impact of the [NH_3_] concentration, i.e., the reaction order, on the intermediate coverage and apparent activation parameters. In fact, we find that the second maximum in activation energy occurs ∼50 mV before the peak potential of the Faradaic NH_3_-to-N_2_ current, beyond which the current and activation parameters decay due to poisoning species. Previously, we hypothesized that the formation of a N_(2)_O or N intermediate during the AOR might capacitively discharge the surface, causing *E*_a_ and log *A* to decrease with overpotential.^21^ Here, our results indicate that already at very low (over)­potentials, the low coverage of specific surface species is sufficient to alter the electron chemical potential and kinetics of the N_2_ formation mechanism.

Although the surface coverage of the various intermediates may be affected by a chemical potential gradient at the interface arising from mass transport limitations, this phenomenon affects the absolute values of the kinetic parameters of the AOR but does not alter their evolution with (over)­potential (see Supplementary Note 7 for more information). In this context, cyclic voltammetry provides information about a dynamically changing interface that can trigger different competing or superimposed processes that result in the total current. Compared to the fast timescales of electron and proton/hydroxide charge transfer (*fs-ps*), cyclic voltammetry provides a new quasi-steady-state condition. However, it should be noted that fast restructuring or other processes at the surface on the timescales of charge transfer itself would constitute processes that could directly couple to processes along reaction coordinate.

The scan-rate dependence of the apparent activation parameters in Figure 4A–B shows that in the intermediate region between ∼0.4–0.55 V_RHE_, both *E*_a_ and log *A* increase with scan rate. As we detail in Supplementary Note 6, this indicates that more excess charge can be built up by rapidly polarizing the surface before the coverage of parallel species starts to depolarize the surface. Above ∼0.55 V_RHE_, both parameters decrease again as the scan rate increases, suggesting that the formation and coverage of the NO species between ∼0.45 and 0.55 V_RHE_ is counteracting or “poisoning” the AOR. This is also consistent with our previous time-resolved Arrhenius analysis that allowed us to track the impact of time-dependent intermediate coverages on a ms-s timescale.^21^

The close correlation between the apparent activation parameters and Δ*E*/log­[NH_3_] in Figure 4 is critical to link the apparent activation parameters extracted from temperature-dependent cyclic voltammetry with the AOR mechanism. Therefore, we also studied the impact of the NH_3_ concentration on the apparent activation parameters (Supplementary Figures 1–3). Figure 5A shows the overpotential-dependent activation energy for 10^–3^– 0.1 M NH_3_. The potentials associated with the minimum (brown star), the maximum *E*_a_ (black circle), and a deactivation point (purple square) all shift to lower values with increasing NH_3_ concentration. Most importantly, the magnitude of the apparent activation parameters is almost identical for the first and second maxima across 10^–2^–10^–1^ M NH_3_ despite different coverage of the intermediates (Figure 3). Together with the results of increasing apparent activation parameters in a region of saturating intermediate coverage with overpotential (Figure 4C), these results evidence that, in general, the apparent activation parameters cannot be reduced solely to the impact of the coverage of some charge-neutral intermediates, as is sometimes implied by some microkinetic models.

**Figure 5:**
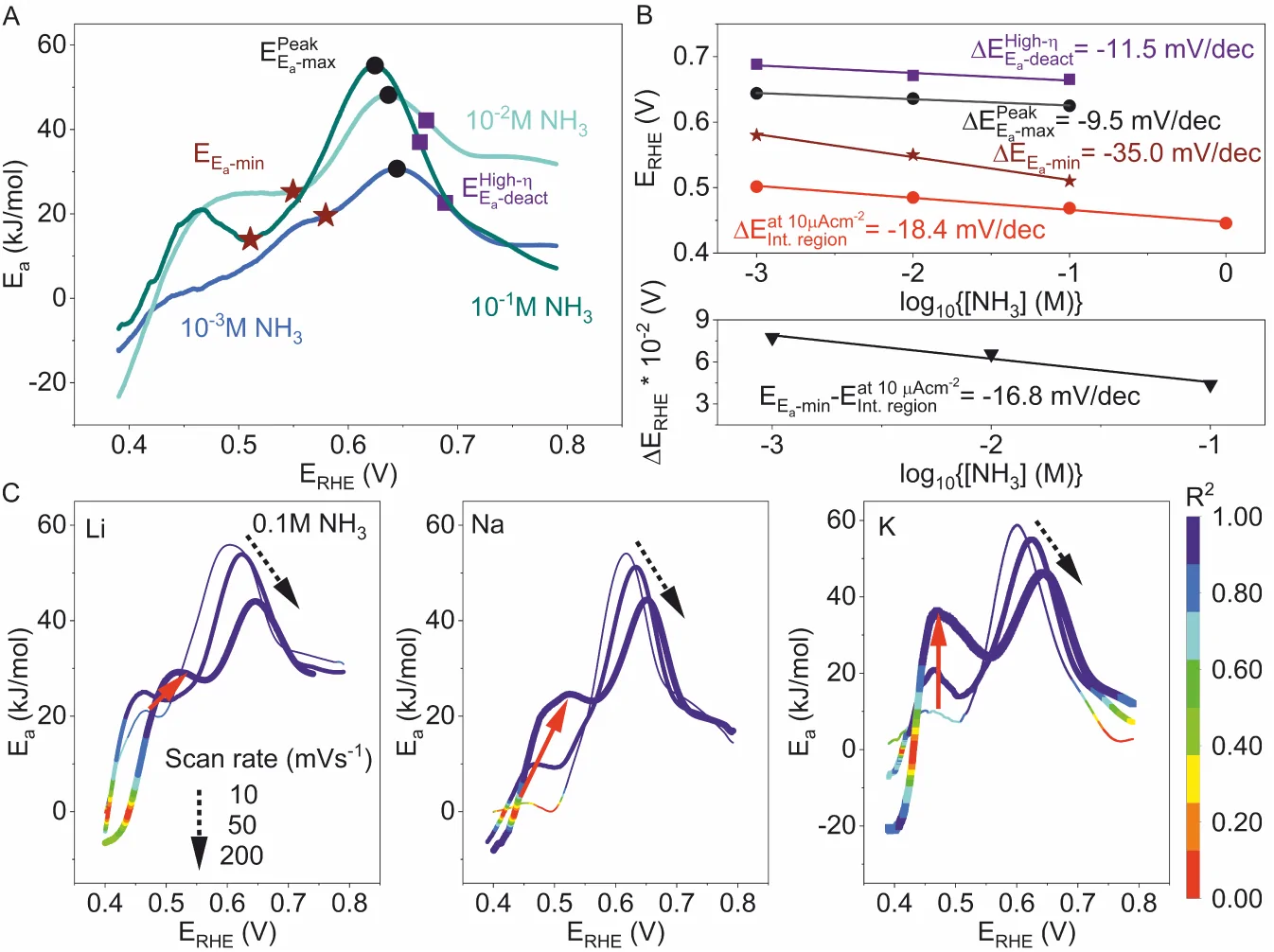
Link of the kinetic descriptors to the potential of zero charge and cation dependence. (**A**) *E*_a_ (left axis, solid lines) and corresponding NH_3_ oxidation currents at 20 °C (right axis, dashed lines) vs *E* at different [NH_3_]. Brown stars mark minima before *E*_a_ rises to its maximum (black circle). Purple squares denote an *E*_a_ that is strongly impacted by deactivation. (**B**) Top: potentials of these characteristic activation energies vs log­[NH_3_] and Δ*E*/log­[NH_3_] from Figure 1B (red circles). Bottom: difference between the potential of minimum *E*_a_ and $\Delta E_{\mathrm{Int. region}}^{10\mathrm{\mu A}{\mathrm{cm}}^{2}}$ /log­[NH_3_]. For all experiments, 0.1 M KOH and a scan rate of 50 mV s^–1^ were used. (**C**) Potential-dependent activation energy (*E*_a_) for 0.1 M LiOH, NaOH, and KOH; scan rates: 10, 50, and 200 mV s^–1^.

As shown in Figure 5B, the shift in the minimum potential (brown stars) with log­[NH_3_] reveals a distinct behavior compared to the other two points. However, when plotting the difference between the shift of the minimum potential and the shift of the onset potential with log­[NH_3_] (red circles), we find a trend very similar to the pztc with log­[NH_3_] (bottom panel of Figure 5B). This suggests that a higher NH_3_ concentration increases the coverage of the parallel species, but this causes a depolarization of the surface by a potential difference that is almost equivalent to the variation in the pztc, which itself depends on [NH_3_]. Consequently, an additional overpotential of comparable magnitude must be applied to recover the initial Δ*E*/log­[NH_3_]. As mentioned earlier, we hypothesize that this counteracting behavior between the formation of the parallel species and the AOR onset is fundamentally limiting AOR activity with increasing NH_3_ concentration. It also suggests that the surface polarization cannot be understood from the pz­(t)­c alone for such complicated reactions. Even in the absence of substantial structural changes in a potential window that largely coincides with the double-layer region of the bare Pt surface, the overpotential-dependent coverage of species modulates the electron chemical potential, and thus the overpotential-dependent formation of excess charge.

To provide further evidence for the influence of interfacial polarization on the ammonia oxidation mechanism, particularly the OH desolvation step, we modulated the interfacial electrostatic environment by varying the electrolyte cations. Similar potential-dependent changes in the kinetic parameters were observed for Li^+^, Na^+^, and K^+^. However, a clear cation-dependent trend emerged when these parameters were examined as a function of scan rate in the potential region most sensitive to changes in surface excess charge below 0.55 V_RHE_.

Figure 5C shows the apparent activation energies obtained for the different cations. A similar trend was observed for log A (see Supplementary Figure 16). Notably, at higher potentials, the cations appear to have a smaller relative impact compared with the initial polarization region. There, the difference between the apparent activation energies measured across different scan rates decreases as the cation size decreases. Further, the potential difference between the inflection points associated with the first deactivation process (red arrows) at the highest and lowest scan rates increases as the cation size decreases. Consequently, for Li^+^, there is a substantial shift in the initial region. Further details on the relationship between the rates of the processes occurring in parallel with the N_2_ evolution and the inflection points are provided in Supplementary Note 6.

Collectively, these results strongly indicate that the oscillations in the apparent activation parameters at lower potentials are closely related to the degree of interfacial polarization, which involves cation rearrangement in the double layer. Compared to small cations, the interfacial environment might be more flexible with larger cations leading to more pronounced oscillations as a function of the scan rate. However, more studies are needed to dissect these dynamic changes in detail, including the general notion that cations should remain largely located in the outer Helmholtz plane. Most importantly, relative changes in the apparent activation parameters are observed for all cations. Therefore, the latter can be considered as modulating the interfacial electrostatic environment but are not the underlying cause of the observations.

Next, we performed surface-enhanced infrared absorption spectroscopy (SEIRAS) to obtain more information about the overpotential-dependent coverage of intermediates.

Figure 6A and B show the spectra recorded on polycrystalline Pt in 0.1 M KOH in the absence of ammonia during a positive-going potential sweep from 0 to 0.4 V_RHE_ at 5 mV s^–1^. The spectra in Figure 6A display a negative band at 1230 cm^–1^ related to the asymmetric stretching vibration of SiO_2_ formed on the Si prism and a positive band at 1163 cm^–1^ likely associated with platinum oxides. Figure 6B shows the spectral region corresponding to the H–O–H bending mode of water.

**Figure 6:**
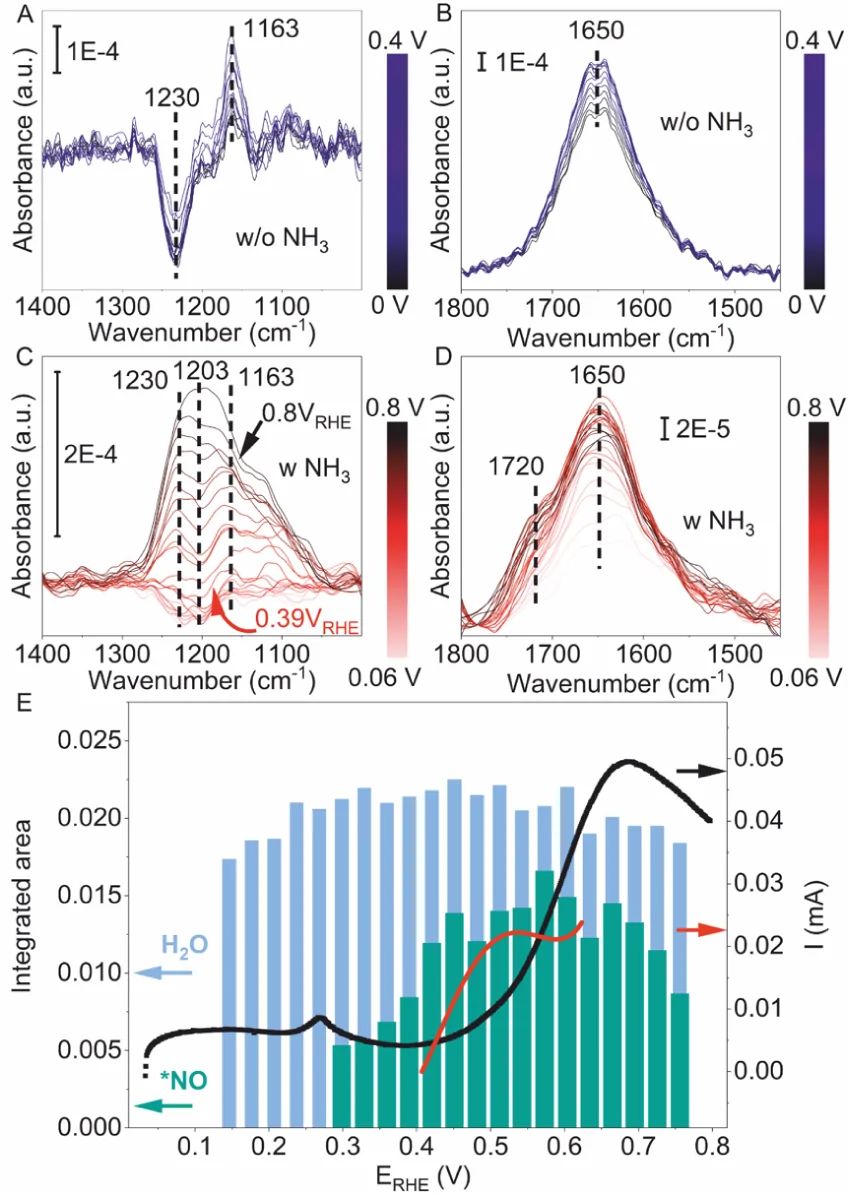
SEIRAS characterization of interfacial species relevant to the kinetic analysis. **(A)** Background spectra recorded between 1400 and 1000 cm^–1^ in the absence of NH_3_ (w/o), showing bands assigned to silica (1230 cm^–1^) from the Si prism and platinum oxides (1163 cm^–1^). (**B**) Background spectra recorded between 1800 and 1450 cm^–1^ in the absence of NH_3_ (w/o), showing the H–O–H bending mode of adsorbed water. (**C**) Spectra recorded in the presence of NH_3_ (w) over the same spectral region as in panel A, revealing multiple bands associated with AOR intermediates. (**D**) Spectra recorded in the presence of NH_3_ (w) over the same spectral region as in panel B, showing a shoulder on the water band assigned to atop *NO. (**E**) Potential-dependent integrated areas of the deconvoluted bands assigned to H_2_O and *NO, plotted together with the simultaneously recorded cyclic voltammogram (black line). The stripping current of the adsorbed species shown in Figure 3 is overlaid for comparison (red line). Experimental conditions: 0.1 M KOH containing 0.1 M NH_3_ at 25 °C; scan rate, 5 mV s^–1^. See Supplementary Figures 22 and 23 for more details.

Figure 6C and D shows the spectral regions equivalent to those presented in Figure 6A and B, respectively, but in the presence of 0.1 M NH_3_. As shown in Figure 6C, the addition of ammonia gives rise to multiple bands, centered around 1203 cm^–1^, which partially overlap with the silica-related modes. These bands become progressively more intense and broader as the potential is swept from 0.06 to 0.8 V_RHE_. On the basis of previous literature assignments, these features are attributed to N_*x*_H_*y*_ reaction intermediates formed during ammonia oxidation.^7,38,39^ The evolution of these bands indicates an increase in the total surface coverage of the reaction intermediates as the applied potential increases up to 0.8 V_RHE_.

In addition, Figure 6D shows a shoulder at approximately 1720 cm^–1^ superimposed on the H–O–H bending band, which we assign to the atop-bound *NO species.^7,39^ As illustrated by the color map between 0.06 and 0.8 V_RHE_, the intensity of this band initially increases with potential and subsequently decreases as the potential approaches 0.8 V_RHE_.

Figure 6E compares the integrated area of the *NO stretching band, obtained after deconvolution from the overlapping H–O–H bending contribution, with the simultaneously recorded ammonia oxidation current (black line). Notably, *NO is already present at very low potentials. This observation reveals the existence of reaction pathways parallel to N_2_ formation at substantially lower overpotentials than might be generally considered. The integrated *NO band area increases with the applied potential, reaches a plateau, and subsequently decreases. This trend is consistent with the charge-derived currents obtained from the coulometric analysis of the potential-step experiments, as shown by the red line and discussed in greater detail in Figure 3 and Supplementary Note 4. On the basis of these results, we attribute the change in the electron’s chemical potential reflected in the kinetic parameters to the parallel formation of *NO. Regarding the bands in Figure 6C assigned to the intermediate N_*x*_H_*y*_ species, a rigorous deconvolution could not be performed due to the complex multiplicity and extensive overlap of the peaks.

## Discussion

Our results support a mechanistic picture of the AOR as an overpotential-dependent process involving multiple parallel reaction pathways. Within the studied potential window, the dominant Faradaic current is associated with NH_3_ oxidation and N_2_ evolution, whose kinetics might be closely linked to the ability to desolvate hydroxide ions inside the double layer. Initially, the N_2_ evolution pathway involves initial deprotonation of adsorbed NH_3_ to NH_2_ in the H^+^/OH^–^ region, followed by at least three distinct steps. Concurrently, between 0.4 and 0.55 V_RHE_, we observe the generation of a species that partially depolarizes the interface. As a result, a larger overpotential is required to build up excess charge again and extract hydroxides that are needed for the progression of the reaction along the N_2_H_2_ pathway toward Faradaic N_2_ evolution. Based on spectroscopic and coulometric results, we attribute this interfacial depolarization to the formation of NO* species and their impact on the potential of zero charge. Compared to N_*x*_H_y_* intermediates, the different chemical nature of NO* likely leads to more pronounced local charge redistribution in the chemical bonds, causing a more pronounced shift in the electron chemical potential.

It is also important to consider the presence of an azide (N_3_^–^) group detected during the AOR via Raman spectroscopy by Zhang et al.,^37^ as well as by SERS by Vidal-Iglesias et al.^38^ This species could, in principle, also induce changes in the electron chemical potential or the pztc. However, a change in these parameters is more plausibly associated with the accumulation of a comparatively stable surface species, while N_3_^–^ acts as a parallel reactive intermediate, as suggested by Vidal-Iglesias et al. On the basis of the spectroscopic evidence presented here, *NO is the most likely species responsible for the effect that we observed.

At potentials above 0.55 V_RHE_, we observe that the apparent activation parameters rise again and that the AOR potentials shift again in a manner similar to that of the pztc with NH_3_ concentration. We hypothesize that this rise in the activation parameters is again associated with the buildup of excess charge and electric fields that are needed to desolvate hydroxide ions and support the progression of the reaction along the N_2_H_2_ pathway toward N_2_ evolution.

Our study also demonstrates that, in general, the apparent activation parameters cannot be reduced solely to the impact of the coverage of some charge-neutral intermediates that push the reaction through different rate-limiting steps, as is sometimes implied by microkinetic models. We observe apparent activation parameters that are almost identical for 10^–2^–10^–1^ M NH_3_, despite a different coverage of the intermediates. Further, we observe *increasing* apparent activation parameters in a region of *saturating* intermediate coverage with overpotential. Further, we see a very close correlation of the potential shifts with the ammonia concentration for the onset and intermediate region that are mirrored by shifts in the potential of zero charge with ammonia concentration. Finally, we see a clear impact of the role of cations on the apparent activation parameters, strongly suggesting a modulation of the double-layer electrostatics. In fact, the cations have a stronger relative impact in the intermediate potential range than the overpotential-dependent coverage itself (which even saturates). Therefore, future microkinetic modeling will be needed to dissect the activation parameters in more detail, but the underlying origin of the AOR multistep reaction cannot be reduced to the sole impact of overpotential-dependent coverage of charge-neutral intermediates.

In conclusion, our results point toward a more comprehensive kinetic picture, where the overpotential-dependent formation of intermediates is closely tied to the stabilization of interfacial excess charge and electric fields that are needed to extract protons and hydroxides at a polarizable interface. This aspect might be quite decisive to understand the very origin of overpotential in many multistep reactions.

## Methods

### Experimental Setup

The electrochemical experiments were conducted in a PTFE cell with the conventional three-electrode configuration, using a platinum wire as the counter electrode and a Luggin capillary to separate the Pt/H_2_ reference electrode from the working solution. Prior to each measurement, the working solution was deoxygenated by bubbling argon for 15 min, and an Ar atmosphere was maintained throughout the experiment. The working electrode was a polycrystalline Pt foil, which was rinsed in 30% nitric acid and flame-annealed before each measurement to ensure a clean and reproducible surface. The electrode potential was controlled by using a Gamry Reference 3000 potentiostat. To control the temperature in every experiment, the electrochemical cell was placed inside a secondary, double-walled bath with temperature controlled via a Julabo CORIO CP-300F thermostat and Teflon-coated thermocouples in the electrolyte.

### Chemicals

All solutions were prepared with ultrapure water obtained from an ELGA PURELAB flex system (resistivity 18.2 MΩ cm, TOC < 1 ppb). The 0.1 M KOH electrolyte was prepared using potassium hydroxide monohydrate (analytical grade, Fisher Chemical), and aqueous ammonia solutions were prepared from a 25% NH_3_ stock solution (Merck). The platinum used as both the counter and working electrodes in this work had a purity of 99.995% (Fisher Scientific). High-purity (5–6 N) gases from Linde were used.

### Charge Analysis in the H^+^ /OH^–^ Region

Cyclic voltammetry experiments were performed at different ammonia concentrations. In particular, the charge associated with the H^+^/OH^–^ adsorption region of the voltammogram was analyzed by integrating the CV data using ammonia concentrations ranging from 10^–5^ to 1 M in alkaline media. To obtain further insight into the effect of the ammonia concentration on the surface charge distribution, CO charge-displacement measurements^39^ were carried out. For these experiments, the electrode was held at the desired potential while a CO gas flow was introduced into the cell, allowing CO to diffuse to the electrode surface. A transient current was recorded, corresponding to the displacement of preadsorbed species by CO poisoning. Once the current decayed to zero, the CO flow was stopped. The solution was then purged with Ar for 15 min to remove dissolved CO, and the CO adsorbed on the surface was stripped to recover the initial state of the electrode (Supplementary Note 2).

The error bars in the voltammograms shown in Figure 2 correspond to the standard deviation of the measured currents. In Figure 2B, the error in the logarithm of the concentration corresponds to the standard deviation of the logarithm of the concentrations, whereas the error along the *Y*-axis represents the half-range associated with a charge variation of ±5 μC/cm^2^. The error in Δ*E*/log­[NH_3_] corresponds to the standard error obtained directly from the graphical representation shown in panel C.

### Potential Jump Measurements

To probe the charge associated with reaction intermediates formed during ammonia oxidation, chronoamperometric measurements were carried out in the potential region where nitrogen evolution takes place (*E* > 0.4 V_RHE_). A potential-pulse protocol was applied in which the electrode potential was stepped from 0.1 V to 0.413, 0.463, 0.513, 0.563, and 0.613 V_RHE_. Each pulse in the nitrogen evolution region was held for 30 s. After each pulse, a stripping current was recorded at 0.1 V_RHE_, corresponding to the cathodic desorption of the intermediates generated during ammonia oxidation.

The measured charge includes capacitive and pseudocapacitive contributions arising from hydrogen adsorption and hydroxide desorption, as well as the capacitive component associated with stepping from the nitrogen-evolution region back to 0.1 V_RHE_. Therefore, two corrections were applied to the stripping charge. First, the charge enclosed in the voltammogram between 0.413 V_RHE_ and 0.1 V_RHE_—containing the capacitive and pseudocapacitive contributions of the H^+^/OH^–^ region— was subtracted. Second, the charge recorded between the nitrogen-evolution potentials and the lowest potential in that region (0.413 V_RHE_) was also subtracted. It is important to note that this latter subtraction may account not only for capacitive contributions within the nitrogen-evolution region but also for charge associated with reversible processes occurring at those potentials. Thus, the resulting net charge can be interpreted as the contribution from the stripping of a species that adsorbs irreversibly in the nitrogen-evolution region (see Supplementary Note 4).

### Arrhenius Analysis

Voltammetric profiles in the N_2_ evolution region were recorded at ammonia concentrations of 10^–3^, 10^–2^, and 10^–1^ M. For each concentration, scan rates of 10, 20, 50, 100, and 200 mV s^–1^ were employed. All measurements were conducted at 10, 15, 20, 25, and 30 °C, and the working solution was saturated with N_2_. From the current densities j, linear regressions were constructed by plotting log_10_ j vs 1/T [K^–1^]. Using the decimal logarithmic form of the Arrhenius equation, log_10_ j = log_10_ A – [*E*_a_/(2.3RT)], the apparent activation energy *E*_a_ was determined from the slope, while the intercept provided the term associated with the pre-exponential factor A (see Supplementary Note 1). To assess the impact of the temperature dependence of the equilibrium potential on our results, we examined its variation over the temperature range of this study (see Supplementary Note 8 and Supplementary Table 8).

### In Situ ATR-SEIRAS Measurements

In situ ATR-SEIRAS measurements were performed using a VERTEX 80v Fourier transform infrared spectrometer (Bruker) equipped with an MCT detector and an A530 reflection unit. The spectra were collected at a spectral resolution of 4 cm^–1^ with a time resolution of 6 s per spectrum.

A home-built single-compartment electrochemical cell was used for the spectroelectrochemical measurements. A reversible hydrogen electrode (RHE) and a platinum foil were used as the reference and counter electrodes, respectively. The working electrode was prepared by electrochemical deposition of platinum onto a chemically gold-coated silicon wafer.

### Au Electrode Preparation

Prior to Au deposition, the Si wafer was mechanically polished by using an alumina suspension. The polished wafer was sequentially ultrasonicated in deionized water, acetone, ethanol, and finally deionized water. The native oxide layer on the Si surface was subsequently removed by immersing the wafer in a 40 wt % NH_4_F solution for 90 s.

A freshly prepared aqueous plating bath containing 16.7 mM NH_4_Cl, 5.0 mM NaAuCl_4_, 75 mM Na_2_SO_3_, 25 mM Na_2_S_2_O_3_, and approximately 1.33 wt % HF was used for the chemical deposition of Au. The pretreated Si wafer was immersed in the plating bath maintained at 55 °C for 4 min. After deposition, the Au-coated Si wafer was thoroughly rinsed with deionized water and dried under Ar gas.

### Electrodeposition of the Pt Film

Electrodeposition was carried out in an aqueous electrolyte containing 0.1 M H_2_SO_4_ and 3 mM H_2_PtCl_6_ by using a conventional three-electrode configuration. The Au-coated Si wafer, a Pt foil, and an Ag/AgCl electrode were used as the working, counter, and reference electrodes, respectively.

The electrode potential was cycled three times between 0.40 and −0.35 V at a scan rate of 50 mV s^–1^. The potential was subsequently held at −0.35 V for 5 min to deposit Pt onto the Au film. After electrodeposition, the Pt-coated electrode was thoroughly rinsed with deionized water and dried under Ar gas. The resulting Pt-coated electrode was used as the working electrode for the in situ ATR-SEIRAS measurements.

## Supplementary Material



## Data Availability

The datasets generated and analyzed during the current study are available in the Zenodo repository, https://doi.org/10.5281/zenodo.22236806.

## Notes

Additional explanation about Arrhenius analysis, charge analysis in the H and OH adsorption region, potential of zero total charge measurements, determination of intermediates via charge analysis of transient potential-step measurements, the impact of diffusional mass transport, temperature-dependent AOR equilibrium potentials, additional data sets of temperature-dependent cyclic voltammograms, overpotential- and scan-rate dependence of apparent activation energy and prefactor, and PZTC and ATR-SEIRAS measurements (PDF)
