# Supplementary material for: Temperature-Dependent Voltammetry Reveals the Potential-Dependent Formation of Reaction Species and Their Impact on Interfacial Excess Charge during the Ammonia Oxidation Reaction on Pt
Source: J Am Chem Soc. 2026 Sep 3;148(36):38631–40. doi: 10.1021/jacs.6c04573 (PMC13587658; doi:10.1021/jacs.6c04573)
Supplement: Supplementary file 1 [file ja6c04573_si_001.pdf]

Supplementary Information

for

Temperature-Dependent Voltammetry Reveals the Potential-Dependent Formation of Reaction Species and their Impact on Interfacial Excess Charge during the Ammonia Oxidation Reaction on Pt

Francisco Sarabia*, Sojung Park, Beatriz Roldan Cuenya, Sebastian Z. Oener*

Department of Interface Science, Fritz-Haber Institute of the Max Planck Society, Berlin, 14195, Germany

*Corresponding authors: sarabia@fhi-berlin.mpg.de, oener@fhi-berlin.mpg.de

Supplementary Note 1

Arrhenius analysis

Arrhenius-type analysis was performed according to

$$j = A(\eta)\, e^{\left(\frac{-\Delta E_a(\eta)}{RT}\right)} \quad (1)$$

with the current density, *j,* and the overpotential-dependent Arrhenius pre-factor, $A(\eta)$, and activation energy, $E_a(\eta)$. For a single-step reaction, $E_a(\eta)$ can inform on the activation enthalpy and log $A(\eta)$ on the activation entropy. Further, $A(\eta)$ also contains at least a reactant concentration term to the power of the reaction order. In this study, we are focusing on $E_a(\eta)$ to reduce the possible impact of other factors. However, even for $E_a(\eta)$, the existence of a multi-step reaction sequence, prevents to directly link the apparent activation parameters obtained from Arrhenius analysis to the activation entropy and enthalpy of a rate-limiting transition state[1–3], but requires micro-kinetic modelling to account for bias dependent degrees of rate control of bias dependent rate limiting intermediates or transition states, as we also recently demonstrated[4].

In particular, for the transient intermediate formation studied with cyclic voltammetry, we need to consider the impact of a changing coverage on the apparent activation energy. However, by correlating the behavior of $E_a(\eta)$ with the excess charge determination via CO displacement, as outlined below, we obtain a strong indication for a root cause related to an electric field dependent double layer and the hydroxide (de)solvation and transfer kinetics. An important role of the electric-field dependent double layer is also further supported by our recent results[5–8] across reactions and conditions, including operando spectroscopy[9], and machine-learning accelerated molecular dynamics and grand canonical DFT by Parrinello and coworkers for the CO_2 reduction reaction[10].

Irrespective of the above, we can take the logarithm of the equation to obtain:

$$log_{10}\ j = log_{10}\, A\,(\eta) - \frac{E_a}{2.3RT} \quad (2)$$

Thus, plotting the current density as a function of 1/T allows extraction of the pre-exponential factor (A) from the intercept and the activation energy (E_a) from the slope.

Supplementary Figures 1-3 present the $\log_{10} A$ and E_a for ammonia concentrations of 0.1 M, 0.01 M, and 0.001 M, respectively, obtained at different scan rates and Supplementary Figure 4-6 show the respective temperature dependent cyclic voltammograms.

Supplementary Note 2

Analysis of the charge in the H and OH adsorption region

The analysis of charge within the H^+/OH^- adsorption region, together with the evolution of their corresponding features in the cyclic voltammograms, is essential for correlating the initial steps of the AOR with the different reaction pathways. Supplementary Figure 7A shows the voltammograms recorded for a polycrystalline Pt surface in alkaline medium (0.1 M KOH) at different ammonia concentrations. The potential window is restricted to the region associated with hydrogen and hydroxyl adsorption–desorption. A striking effect is observed in the dependence of the charge distribution and peak positions on ammonia concentration. The presence of ammonia in the electrolyte shifts the entire charge toward lower potentials. When the total charge associated with the voltammogram is plotted against log[NH_3] (Supplementary Figure 7B), it becomes evident that, initially, the addition of NH_3 decreases the charge relative to the blank (horizontal line centered at 210 μC cm^{-2}). Upon further increasing the concentration, the voltammetric charge rises until it finally decreases beyond 0.1 M [NH_3].

These oscillations can be attributed to the adsorption of NH_3 and the subsequent formation of the first reaction intermediate in the multi-step sequence. Previous infrared spectroscopic studies on polycrystalline Pt have identified NH_2 species as the main intermediate formed within this potential window.[11] Therefore, the initial drop in charge relative to the blank arises from a reduced availability of active sites for H and OH adsorption due to the formation of NH_2 species. In particular, it is reasonable to assume that NH_3 is the first adsorbed species, which progressively transforms into NH_2 as the potential increases.[12] At higher surface coverage of NH_3, the fraction of the total voltammetric current involved in NH_2 formation increases at more positive potentials, leading to the observed increase in charge with ammonia concentration.

It is important to note that the overpotential required to convert all NH_3 species into the first reaction intermediate may increase with surface coverage due to lateral interactions among adsorbed molecules. If NH_3 itself does not displace adsorbed hydrogen, this would explain why the charge at low NH_3 concentrations is smaller than in the blank experiment. In this case, NH_2 species would form at very low potentials, displacing hydrogen without being significantly detected in the voltammogram. Finally, since increasing NH_3 concentration shifts the total charge toward lower potentials, part of the charge might be masked by the onset of hydrogen evolution, which would account for the lower integrated charge at higher concentrations. However, as shown later by the CO charge displacement experiments, the degree of surface blocking by reaction intermediates is considerably greater.

The overall shift of the total charge toward lower potentials originates from a change in the chemical potential of the catalyst surface. This phenomenon is illustrated in Supplementary Figure 7C, which shows the variation of the total charge with potential at different NH_3 concentrations (see Section 2 for details). The potential at which the curve crosses the x-axis corresponds to the potential of zero total charge (pztc). The evolution of this parameter with NH_3 concentration provides direct information on the change of surface chemical potential, since it is closely related to both, the work function and the excess charge at the catalyst surface.

Plotting the pztc as a function of log[NH_3] reveals a linear trend with a slope of 19 mV dec^{-1} (Supplementary Figure 7D). This value closely matches the potential shift (22 mV dec^{-1}) observed for the small pre-peak appearing just before the double-layer region in the voltammograms. Assuming that this pre-peak corresponds to H and OH adsorption on Pt(100) facets, the presence of N-containing adsorbates decreases the work function of the surface,

shifting these processes toward lower potentials. Consequently, as the NH_3 concentration increases, the coverage of nitrogenated species grows, inducing a change in the surface chemical potential that affects all subsequent interfacial processes.

To quantify the charge associated with hydrogen and hydroxyl adsorption, a specific criterion was established, as the overall charge distribution shifts toward lower potentials. The integrated charge was obtained from the cyclic voltammogram starting from the double-layer region up to the potential range where hydrogen adsorption begins to overlap with hydrogen evolution, which was defined as the lower potential limit. This point was identified by the change in curvature observed in the negative-going scan at approximately $0.1V_{RHE}$ (Supplementary Figure 8). Here we note that this change in curvature already overlaps with the HER for 1M NH_3. Therefore, we just followed the trend observed at the other ammonia concentrations to define the lower limit for 1M NH_3. Red arrows indicate the corresponding lower potential limit for each ammonia concentration.

The contribution from the double-layer region was subtracted from the total integrated charge. The capacitive component is illustrated for the cyclic voltammogram recorded in 1 M NH_3 (orange line), where the shaded area (light red) represents the extrapolated double-layer region (dark red) extended to the lower potential limit.

Supplementary Note 3

Determination of the potential of zero total charge (pztc)

To quantify how the formation of the earliest intermediates affects the overall surface charge, CO charge-displacement experiments were performed. This approach enables an indirect evaluation of how the interfacial electric field evolves during the formation of specific adsorbed species. The total charge vs. potential curves shown in Supplementary Figure 9 were obtained by integrating the voltammetric response, using as reference the CO displacement charge recorded at the lower potential limit (0.09-0.1 V_{RHE} in this work). The integration was performed according to Eq. (3):

$$q(E) = q(E^{dis}) + \int_{E^{dis}}^{E} \frac{j}{v}\, dE \qquad 3$$

where v represents the scan rate, j is the voltammetric current density, E^{dis} denotes the potential at which the CO displacement occurs, and $q(E^{dis})$ is the total charge.[13] The pztc is determined from the point where the total charge curves intersect the x-axis.

It should be emphasized that $q(E^{dis})$ does not coincide exactly with the measured CO displacement charge, since the interfacial charge of the CO-covered electrode, q_{CO}, must also be considered. Therefore:

$$q(E^{dis}) = q_{CO} - q_{dis} \qquad 4$$

The term q_{dis} corresponds to the experimentally obtained displacement charge, while q_{CO} can be estimated from the differential interfacial capacitance (C_{diff}) measured in the potential region close to the CO displacement, where no faradaic current from CO oxidation is observed. This correction is expressed as:

$$q_{CO} = C_{diff}(E^{dis} - E_{pzc,CO}) \qquad 5$$

The C_{diff} values are 30 µF cm^{-2} at pH 13 on Pt(poly) with and without NH_3 in solution. Reported values for the potential of zero charge for the CO-covered surface ($E_{pzc,CO}$) place it near 1 V_{SHE} for Pt(111), as shown by Cuesta and Weaver[14,15]. Boronat-González *et al.* obtained comparable results, with $E_{pzc,CO} \sim$ 1.0 V_{SHE} for Pt(111) and slightly lower values of 0.9 V_{SHE} and 0.8 V_{SHE} for Pt(100) and Pt(110), respectively[16]. Based on these precedents, an $E_{pzc,CO}$ value of 1 V_{SHE} was adopted for the CO-covered polycrystalline Pt electrode in this work.

The blue circles and orange stars shown for the blank and 1 M NH_3 respectively in Supplementary Figure 9, correspond to total charge values obtained directly from CO charge-displacement experiments and from the correction of the CO charge at the corresponding potentials. In other words, these data points result from applying equations 4 and 5 to the charge values derived from the transient currents presented in Supplementary Figures 10-11. As can be seen, these charge values fit remarkably well with the q_{total}–E curve, confirming that the current responses recorded in both, the charge-displacement and voltammetric measurements are reliable. However, for 1 M NH_3, the pztc deviates from the linear trend of 19 mV dec^{-1} observed at lower concentrations. This deviation is attributed to a loss of total charge associated with the higher surface coverage of reaction intermediates.

Supplementary Note 4

Detection of intermediates via potential-step measurements

A potential-step program was applied between the intermediate and "NH_3 oxidation" region (>0.4 V_{RHE}) and the stripping region. In this procedure, the potential was held constant for 30 s within the NH_3 oxidation range, followed by recording at 0.1 V_{RHE} the current associated with the stripping of adsorbed species formed during the reaction.

To extract the charge associated with the adsorbed intermediates, the capacitive and pseudocapacitive contributions from reversible adsorption–desorption were subtracted from the total current recorded during the stripping step at 0.1 V_{RHE}. The corresponding expression is:

$$q_{intermediates} = q_{stripping} - q_{H-OH} - q_{E_{AOR}-0.413V} \quad 6$$

where $q_{stripping}$ is the total charge recorded upon stepping from a potential above 0.4 V_{RHE} to 0.1 V_{RHE}, q_{H-OH} represents the charge contribution from H and OH adsorption processes when stepping from 0.4 V_{RHE} to 0.1 V_{RHE}, and $q_{E_{AOR}-0.413V}$ accounts for capacitive and reversible processes occurring between the ammonia oxidation potentials and 0.413 V_{RHE}.

Supplementary Figures 13-14 show the currents recorded at 0.1 V_{RHE} and 0.413 V_{RHE}, respectively, after stepping from different potentials within the AOR region. Supplementary Tables 4-5 list the corresponding values of $q_{stripping}$ and $q_{E_{AOR}-0.413V}$. The geometric area of the electrode used was 0.521cm^2.

The main presents representative current density values obtained by dividing the fitted charge curve (connecting the data points in Figure S15) by the time (30 s) required to reach a specific coverage of the adsorbed species formed during ammonia oxidation at each potential.

Supplementary Note 5

Evaluation of the reaction order and reaction mechanism

To compare the potential-dependent variation of the kinetic parameters with possible changes in the reaction mechanism, a reaction order analysis was performed. The process can be expressed by the simplified rate equation:

$$j = k\,[NH_3]^{\alpha}[OH^-]^{\beta} \quad (7)$$

where k is the rate constant, which encompasses the effects of lateral interactions between reaction intermediates, all other intermediates (with a bias dependent coverage) besides NH_3 in the multi-step sequence, and bias dependent changes in the activation enthalpy and entropy of the rate-limiting transition states. Taking the logarithm gives:

$$log_{10}j = log_{10}\left(k + [OH^-]^{\beta}\right) + \alpha log_{10}[NH_3] \quad (8)$$

Hence, the parameter α can be obtained from the slope of the plot of $log_{10}\,j$ versus $log_{10}\,[NH_3]$. Supplementary Figure 19 shows the dependence of logj/log[NH_3] on potential at different scan rates and at 20°C and 30°C, respectively. The current densities used for the reaction order analysis are shown in Supplementary Figure 20.

Supplementary Note 6

Relationship between the evolution of the kinetic parameters and the rates of parallel reactions

The shift of the inflection points in the kinetic parameters with scan rate across different NH_3 concentrations indicates the presence of different processes operating at different rates. In **Supplementary Figure 21A**, we plot the minimum of the activation energy (brown star in Figure 5A) against log[NH_3] and observe that the slope is susceptible to the scan rate. Simplified, in this region the speed of the scan rate impacts the formation of the parallel species and, thus, the shift with potential. In contrast, at the potential corresponding to the maximum activation energy in the Faradaic region (**Supplementary Figure 21B)**, the slopes remain similar, regardless of the scan rates, indicating that the involved adsorbed species processes are much faster than could be accessed in this range of scan rates. We note, that substantially higher scan rates impacted the reproducibility of the temperature-dependent cyclic voltammetry. Finally, in **Supplementary Figure 21C** we show the variation log j/log[NH_3] or reaction order as a function of potential. As shown, for the three scan rates (50 100 and 200 mV s^{-1}) the overpotential-dependent reaction order qualitatively mirrors the evolution of the pre-exponential factor (**Supplementary Figure 21D**), but with distinct relative magnitudes of the minima and maxima. This further supports that the pre-exponential factor cannot be simply explained by a changing and potential-dependent NH_3 concentration on the surface, but contain important kinetic information on the underlying rate constant.

Supplementary Note 7

Impact of the diffusion mass transport in the kinetic parameters

The ammonia oxidation reaction operates under kinetic regime influenced by both diffusive mass transport and electron transfer[17]. Electrochemical systems of this type exhibit a characteristic relationship between the peak current in the voltammogram and the square root of the scan rate. In particular, for irreversible systems, this relationship is given by[18]:

$$i_P = (2.99 \cdot 10^5) A C D^{\frac{1}{2}} v^{\frac{1}{2}} \alpha^{\frac{1}{2}} \quad (9)$$

As shown in Supplementary Figure 24A, for the different temperatures, plotting the measured peak current against the square root of the scan rate yields a straight line with a near-zero intercept. If the peak current were governed exclusively by diffusion, the temperature dependence of the slope ($\Delta I_P/\Delta\sqrt{v}$) would be expected to follow a trend similar to that of the temperature-dependent diffusion coefficient. However, Supplementary Figure 24B shows a deviation in the evolution of the slopes with temperature. This suggests that the maximum current reached in each experiment is not determined solely by mass-transport limitations, but rather by a parallel catalyst-poisoning mechanism.

On the other hand, the reagent consumption rate involved in the CV between $0.4V_{RHE}$ and the peak potential can be compared with the hypothetical case of a purely diffusion-controlled system, for which the charge density is described by:

$$q\ (C\ m^{-2}) = j(C\ s^{-1} m^{-2}) t(s) = \frac{\mathrm{F n D_{NH_3}} [NH_3]}{\delta}\ t \quad (10)$$

Where D_{NH_3} ($m^2\ s^{-1}$) is the diffusion coefficient of ammonia, $[NH_3]$ (mol/l) is the ammonia concentration in the bulk solution, $\delta = \sqrt{\pi D_{NH_3} t}$ and t is the time required to reach the peak potential in the CV. The diffusion coefficient can be estimated using the Stokes–Einstein equation:

$$D_{NH_3} = \frac{k_B T}{6\pi\eta r} \quad (11)$$

Using the diffusion coefficient for ammonia ($2.1\text{x}10^{-5}\ m^2/s$) and the viscosity of water reported in the literature, its value at different temperatures can be estimated.

Based on these approximations and choosing 0.001M NH_3 at 10mV/s as a representative conditions, we find that the total charge passed up to the peak potential for for 30ºC and 10ºC is 22% and 14%, respectively, of the current required to reach diffusion-limited conditions. This further supports the presence of a kinetic control at the catalyst surface, without reaching the diffusion limited current. Therefore, we assume a steady state in the potential window at every single potential step of the CV where:

$$j = nFD_{NH_3}(T) \left[\frac{\partial [NH_3]}{\partial x}\right]_{x=0} (T) = nFk_{rls}\theta_{NH_2} a_{OH} \quad (12)$$

In this case we also assume the following boundary conditions:

$$[NH_3](0,t) = 0 \quad 13$$

$$\lim_{x \to \infty} [NH_3] = [NH_3]^{Bulk} \quad 14$$

$$[NH_3](x,0) = [NH_3]^{Bulk} \quad 15$$

Because both the diffusion coefficient and kinetic reaction are temperature dependent, the diffusion layer thickness differs among the temperature of the study. Consequently, the spatial concentration profile of ammonia between the surface and the bulk also varies with temperature. This, in turns, leads to a temperature-dependent chemical potential gradient. In addition, in the absence of imposed convention, the chemical potential changes during the potential sweep. Thus, we can consider the following equation:

$$i = A\, exp\, [-\frac{\Delta E_a(E_0 + vt) + \Delta G(T,t,x)}{RT}] \quad 16$$

Where $\Delta E_a(E_0 + vt)$ is the potential dependent activation energy, vt is the product of the scan rate and time, and $\Delta G(T,t,x)$ is the contribution to the variation in activation energy arising from the chemical-potential gradient at different temperatures. This depends on $-RTlna_{NH_3}$, where a_{NH_3} is the activity of ammonia species in solution at each instant of the potential sweep, temperature and distance from the catalyst surface.

Applying the logarithm gives:

$$logi = \log A - [\Delta E_a(E_0 + vt) - RTlna_{NH_3}\,(T,t,x)]\frac{1}{2.303RT} \quad 17$$

Differentiating with respect to $1/T$,

$$\frac{\partial logi}{\partial(1/T)} = \frac{1}{2.303}\left[-\frac{\Delta E_a(E_0 + vt)}{R} + \frac{\partial lna_{NH_3}\,(T,t,x)}{\partial(1/T)}\right] \quad 18$$

It is important to note that the activity depends on the variation of concentration with distance from the electrode for a given thickness of the time-dependent diffusion layer. Therefore, to estimate $\frac{\partial lna_{NH_3}}{\partial(1/T)}$, it is necessary to establish a criterion to define the diffusion layer and, in addition, to specify the value of x at which the change in chemical potential is to be evaluated. Given that the solution to the second term of eq 12 is[18]:

$$\frac{\Delta[NH_3]}{\Delta x} = \frac{[NH_3]^{Bulk} - [NH_3](0,t)}{\sqrt{\pi D_{NH_3} t}} \quad 19$$

Where $[NH_3](0,t) \approx 0$ y $\delta = \sqrt{\pi D_{NH_3} t}$.

Therefore, for each temperature, we can approximate the variation in the concentration gradient at each time point of the voltammetry, where:

$$t = \sum_{t_i=0} \frac{E_n - E_{n-1}}{v} \qquad (20)$$

Here, $E_n - E_{n-1}$ is the potential difference between consecutive points recorded in the voltammogram, and v is the scan rate. By defining an approximate distance x from the outer Helmholtz plane (1 nm), we can plot $ln\{([NH_3]^{Bulk}/\delta) \cdot x\}$ vs $1/T$. From the slope, we obtain a value of $\frac{\partial ln a_{NH_3}}{\partial(1/T)}$ for each point of the CV, which can be used to approximate the correction to the kinetic parameters.

Supplementary Figure 26 shows $\frac{\partial ln a_{NH_3}}{\partial(1/T)}$ vs E_{RHE} for 0.1M NH_3 at 50 mVs^{-1}. Using these values to correct the activation energy (Supplementary Figure 27), we observe that diffusion effects alter the *x*-dependent absolute value of the kinetic parameter, but do not change its potential-dependent trend.

Supplementary Note 8

Temperature correction of the equilibrium potential for the AOR

Since the thermodynamic equilibrium potential of a reaction depends on temperature, its temperature dependence should be corrected to ensure that all temperature-dependent measurements used in the kinetic analysis are compared at the same driving force.

In the present case, literature data were used for the temperature dependence of the standard thermodynamic potential ($\partial E^{\circ}/\partial T$) for the reduction of N_2 to NH_3 in acidic media[19]. Because our study was conducted under alkaline conditions, an appropriate temperature correction requires a relationship between the standard reaction potentials in acidic and alkaline media. To this end, the reduction of nitrogen to ammonia in alkaline media can be written as the sum of the corresponding reaction in acidic media and water dissociation:

$$6e^{-} + 6H^{+} + N_2 \rightarrow 2NH_3 \quad (21)$$

$$6 \times \quad H_2O \rightarrow H^{+} + OH^{-} \quad (22)$$

$$6e^{-} + 6H_2O + N_2 \rightarrow 2NH_3 + 6OH^{-} \quad (23)$$

The total Gibbs free-energy change for the overall process is:

$$\Delta G^{\circ}_{Alk} = \Delta G^{\circ}_{Ac} + 6\Delta G^{\circ}_{H_2O} \quad (24)$$

Relating the Gibbs free-energy change to the standard potential and the water dissociation constant gives:

$$-6F\Delta E^{\circ}_{Alk} = -6F\Delta E^{\circ}_{Ac} - 6RT lnK_w \quad (25)$$

After simplification, the following relationship is obtained between the standard potentials in alkaline and acidic media:

$$\Delta E^{\circ}_{Alk} = \Delta E^{\circ}_{Ac} + \frac{RT}{F} lnK_w \quad (26)$$

Accordingly, the Nernst equation for the reaction in alkaline media is:

$$\Delta E^{SHE}_{Alk} = \Delta E^{\circ}_{Alk} + \frac{RT}{6F} ln \frac{a^6_{H_2O} a_{N_2}}{a^2_{NH_3} a^6_{OH^-}} \quad (27)$$

Substitution of eq 26 into eq 27 gives the relationship between the equilibrium potential in alkaline media and the standard potential in acidic media:

$$\Delta E_{Alk}^{SHE} = \Delta E_{Ac}^{\circ} + \frac{RT}{F} lnK_w + \frac{RT}{6F} ln \frac{a_{H_2O}^6 a_{N_2}}{a_{NH_3}^2 a_{OH^-}^6} \quad 28$$

To convert from the SHE scale to the RHE scale, the relationship between the two reference scales is written as:

$$2e^- + 2H^+ \leftrightarrow H_2 \quad 29$$

$$\Delta E_{RHE-SHE} = \frac{RT}{2F} a_{H^+}^2 = \frac{RT}{F} a_{H^+} = \frac{RT}{F} lnK_w - \frac{RT}{F} a_{OH^-} \quad 30$$

Conversion from the SHE to the RHE scale requires taking the difference between the reaction potential measured versus the standard hydrogen electrode and the potential given by eq 30:

$$\Delta E_{Alk}^{RHE} = \Delta E_{Alk}^{SHE} - \Delta E_{RHE-SHE} \quad 31$$

The Nernst equation on the RHE scale therefore becomes:

$$\Delta E_{Alk}^{RHE} = \Delta E^{\circ}{}_{Ac} + \frac{RT}{F} lnK_w + \frac{RT}{6F} ln \frac{a_{H_2O}^6 a_{N_2}}{a_{NH_3}^2 a_{OH^-}^6} - \left[\frac{RT}{F} lnK_w - \frac{RT}{F} a_{OH^-}\right] \quad 32$$

Expanding this expression gives:

$$\Delta E_{Alk}^{RHE} = \Delta E^{\circ}{}_{Ac} + \frac{RT}{6F} ln \frac{a_{H_2O}^6 a_{N_2}}{a_{NH_3}^2} \quad 33$$

Here, the activity of water (a_{H_2O}) is taken as unity because water is a pure liquid. In addition, because N_2 and NH_2 are gases and their partial pressures were not controlled in this study, whereas their dissolved concentrations were, the partial pressures must be converted into concentrations using Henry's law. Thus, the activity of a gaseous species is given by:

$$a_g = f_g \frac{p_g}{p_g^{\circ}} = f_g \frac{C_g / k_H}{p_g^{\circ}} \quad 34$$

where a_g is the activity of the gas, p_g is its partial pressure, p_g° is the standard pressure, C_g is its concentration and k_H is the Henry's law constant.

Equation 33 than becomes:

$$\Delta E_{Alk}^{RHE} = \Delta E^{\circ}{}_{Ac} - \frac{RT}{6F} lnk_H^{N_2} + \frac{RT}{6F} ln\left(k_H^{NH_3}\right)^2 + \frac{RT}{6F} ln\frac{C_{N_2}}{C_{NH_3}^2} \quad 35$$

Differentiation of eq 35 with respect to temperature gives the temperature coefficient for the reduction of nitrogen to ammonia:

$$\frac{\partial \Delta E_{Alk}^{RHE}}{\partial T} = \frac{\partial \Delta E^{\circ}{}_{Ac}}{\partial T} - \frac{R}{6F} lnk_H^{N_2} - \frac{RT}{6F}\frac{\partial lnk_H^{N_2}(T)}{\partial T} + \frac{R}{3F} lnk_H^{NH_3} + \frac{RT}{3F}\frac{\partial lnk_H^{NH_3}}{\partial T} + \frac{R}{6F} lnC_{N_2} + \frac{R}{6F}\frac{\partial lnC_{N_2}}{\partial T} - \frac{R}{3F} lnC_{NH_3} \quad 36$$

Because the temperature coefficient for a reduction process is equal in magnitude and opposite in sign to that of the corresponding oxidation process,

$$\frac{\partial \Delta E_{red}}{\partial T} = -\frac{\partial \Delta E_{ox}}{\partial T} \quad 37$$

the temperature dependence of the thermodynamic potential for ammonia oxidation on the RHE scale is:

$$\frac{\partial \Delta E_{Alk}^{RHE}}{\partial T} = -\frac{\partial \Delta E^{\circ}{}_{Ac}}{\partial T} + \frac{R}{6F} lnk_H^{N_2} + \frac{RT}{6F}\frac{\partial lnk_H^{N_2}(T)}{\partial T} - \frac{R}{3F} lnk_H^{NH_3} - \frac{RT}{3F}\frac{\partial lnk_H^{NH_3}}{\partial T} - \frac{R}{6F} lnC_{N_2} - \frac{R}{6F}\frac{\partial lnC_{N_2}}{\partial T} + \frac{R}{3F} lnC_{NH_3} \quad 38$$

Because all parameters at each temperature can be obtained from the literature[20,21], eq 38 does not need to be evaluated explicitly to correct for the temperature dependence of the equilibrium potential. A temperature of $25°C$ was used as the reference, and the change in equilibrium potential relative to this temperature was calculated. Thus, eq 35 was evaluated at each temperature, and the corresponding value was referenced to that at $25°C$. For the difference in the $\Delta E^{\circ}{}_{Ac}$ term in eq 35 between a temperature T and $25°C$, the literature-reported temperature coefficient for the anodic process in acidic media ($\partial E^{\circ}/\partial T = 0.342 mV/K$)[19] was multiplied by the corresponding temperature difference. As shown in Supplementary Table 8, the change in the equilibrium potential for ammonia oxidation under all investigated conditions, relative to 25 °C, is negligible.

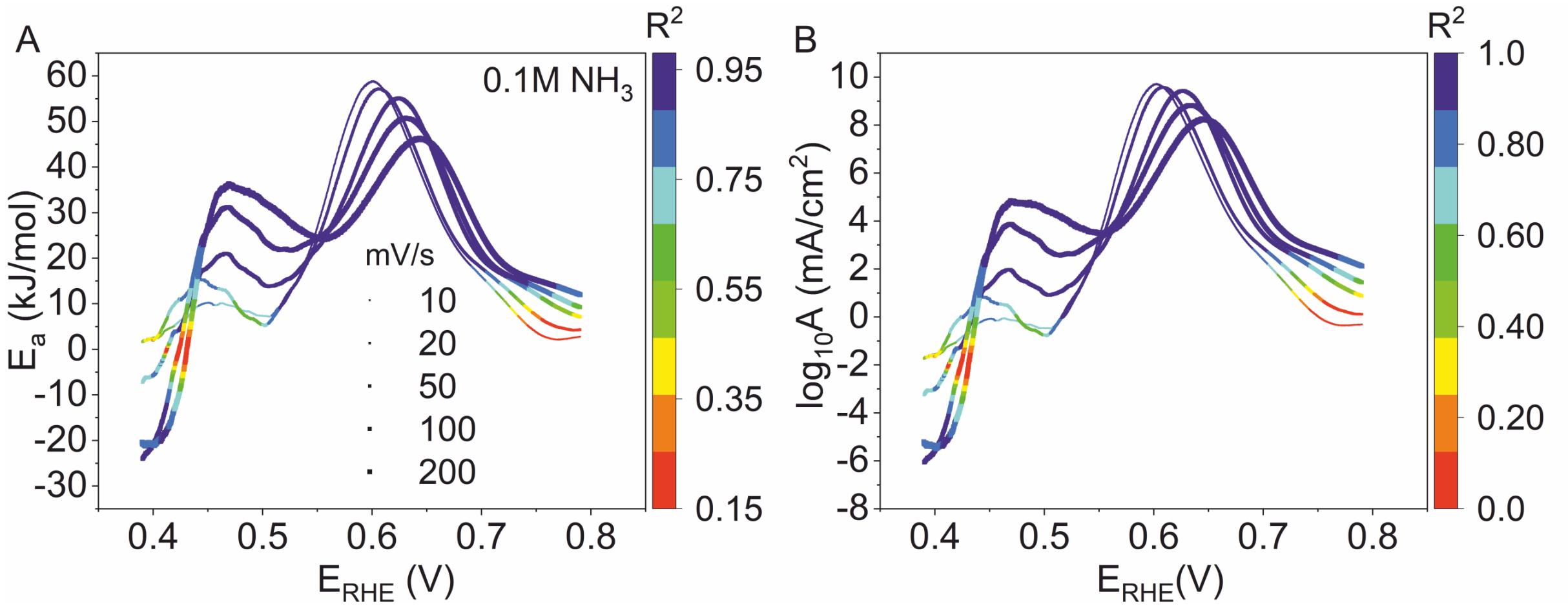

Supplementary Figure 1. Bias and scan-rate dependence of apparent activation energy (E_a) and Arrhenius pre-factor (log A). Dependence of the activation energy **(A)** and pre-exponential factor **(B)** on potential for 0.1 M NH_3 at different scan rates.

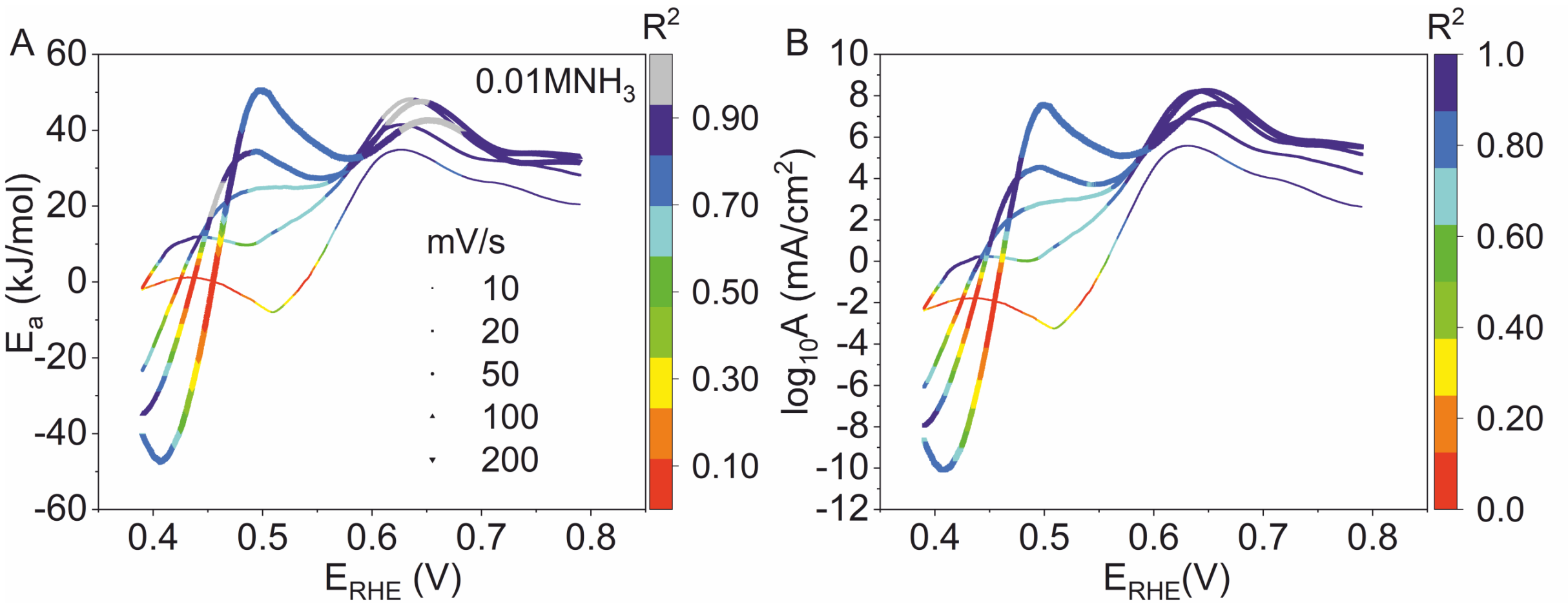

Supplementary Figure 2. Bias and scan-rate dependence of apparent activation energy (E_a) and Arrhenius pre-factor (log A). Dependence of the activation energy **(A)** and pre-exponential factor **(B)** on potential for 0.01 M NH_3 at different scan rates.

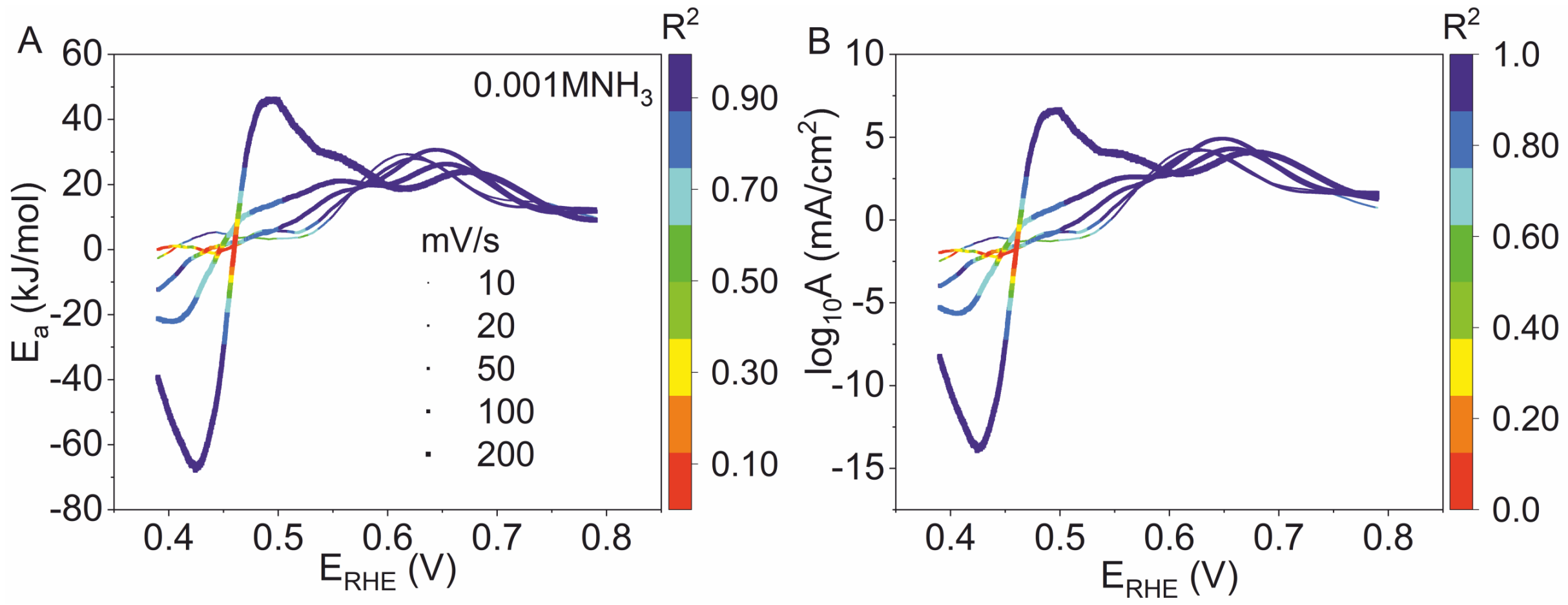

Supplementary Figure 3. Bias and scan-rate dependence of apparent activation energy (E_a) and Arrhenius pre-factor (log A). Dependence of the activation energy **(A)** and pre-exponential factor **(B)** on potential for 0.001 M NH_3 at different scan rates.

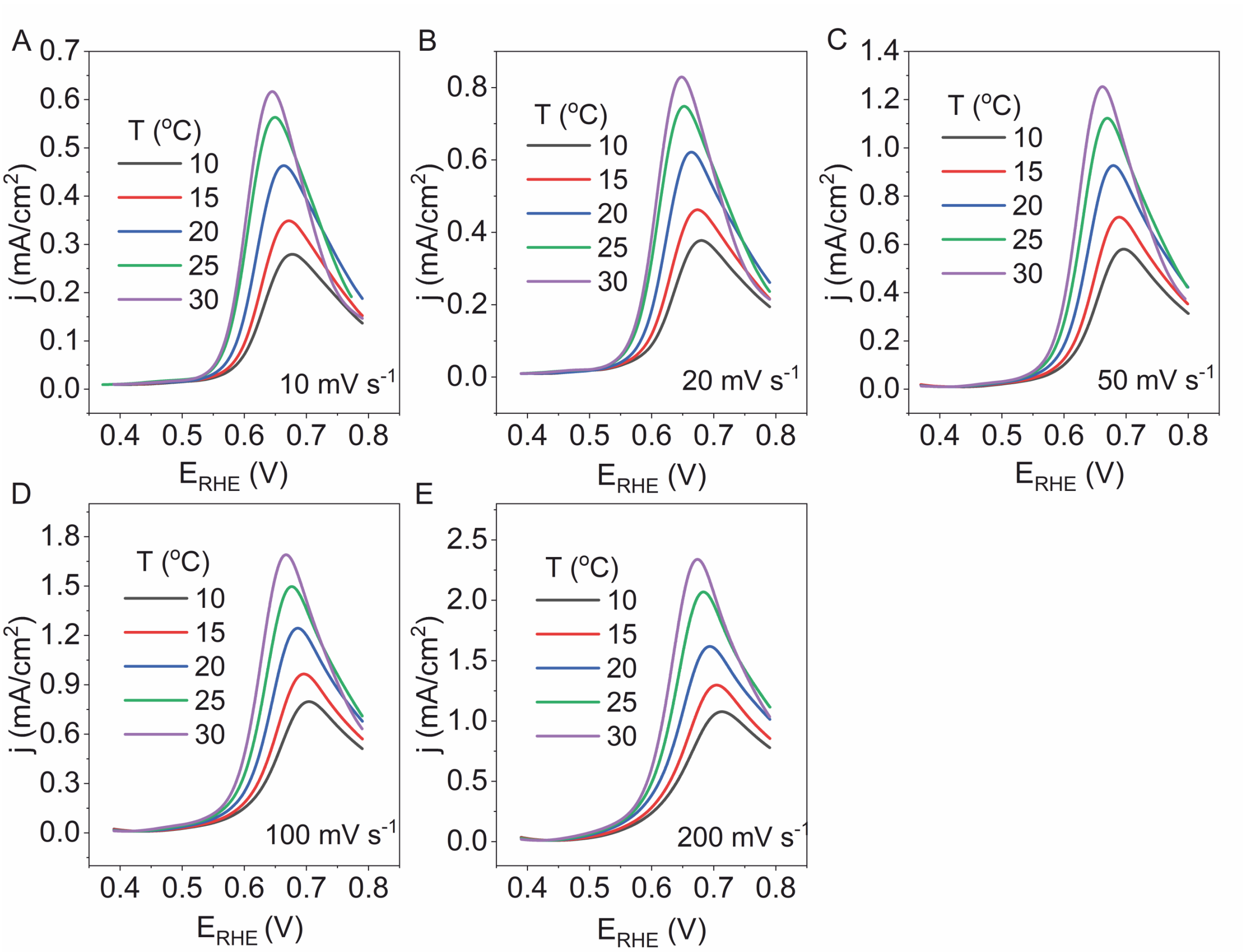

Supplementary Figure 4. Positive going scan of the CVs in the N_2 evolution region during AOR. Current densities used to extract the kinetic parameters shown in Supplementary Figure **1**. 0.1M KOH + 0.1M NH_3.

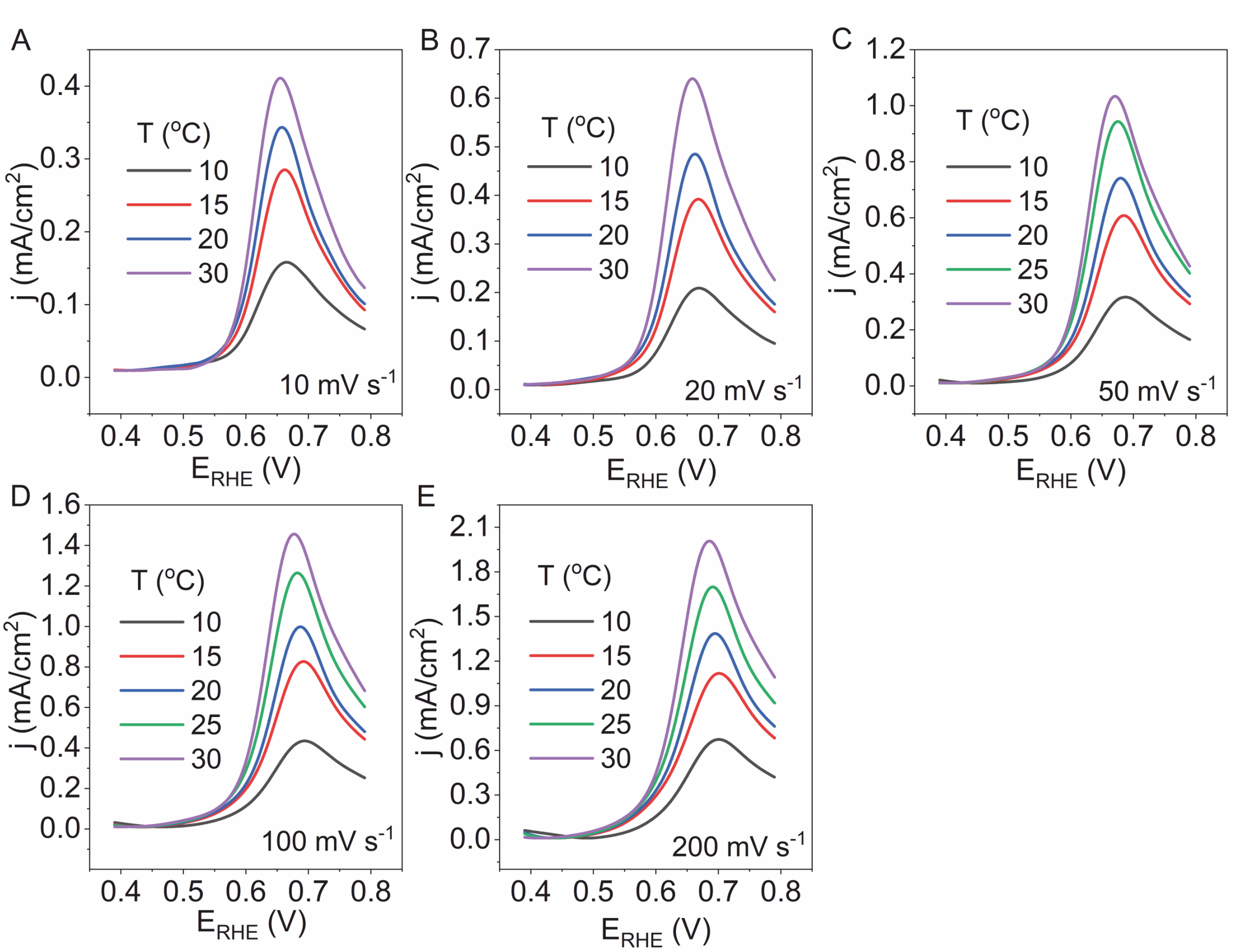

Supplementary Figure 5. Positive going scan of the CVs in the N_2 evolution region during AOR. Current densities used to extract the kinetic parameters shown in Supplementary Figure 2. 0.1M KOH + 0.01M NH_3.

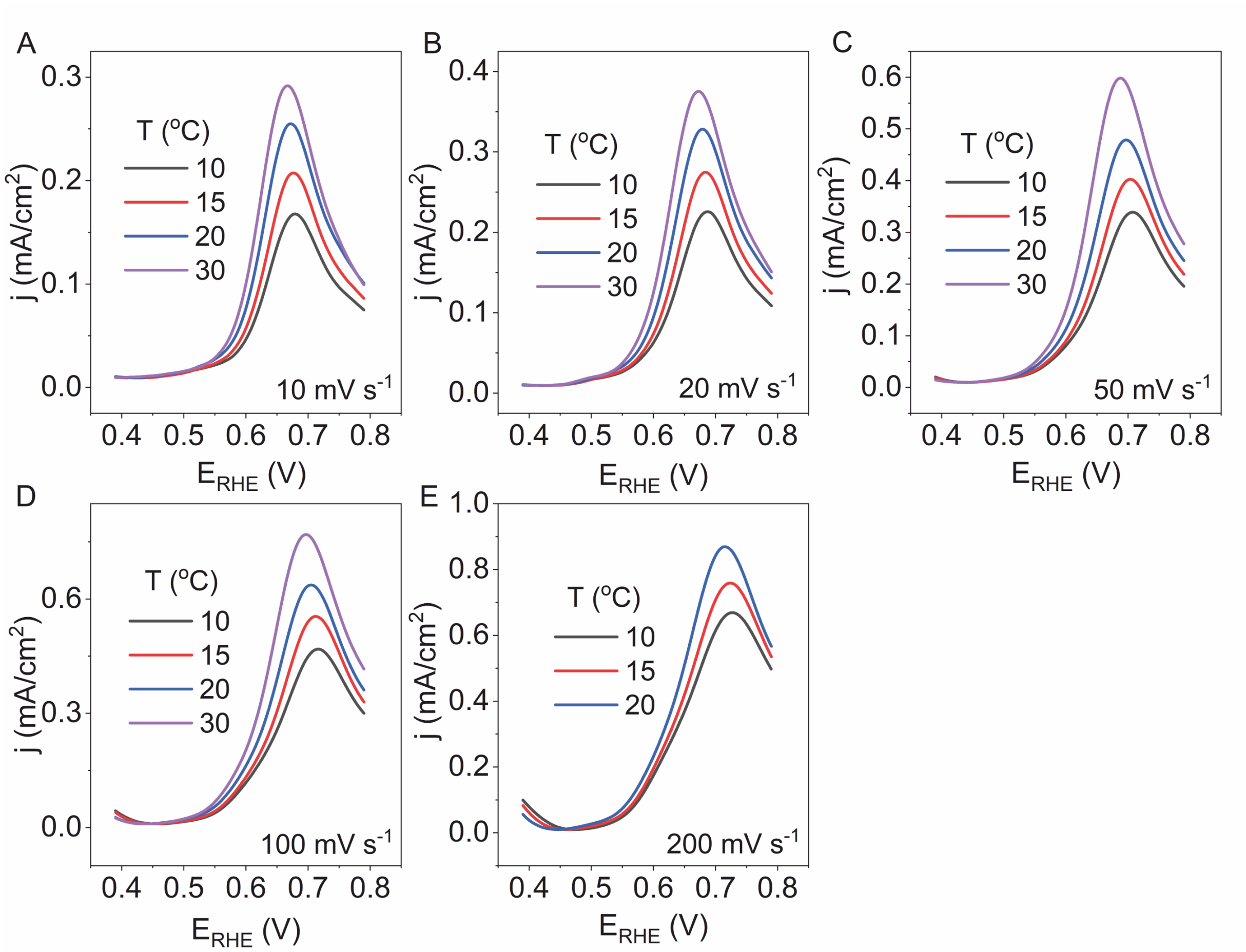

Supplementary Figure 6. Positive going scan of the CVs in the N_2 evolution region during AOR. Current densities used to extract the kinetic parameters shown in Supplementary Figure 3. 0.1M KOH + 0.001M NH_3.

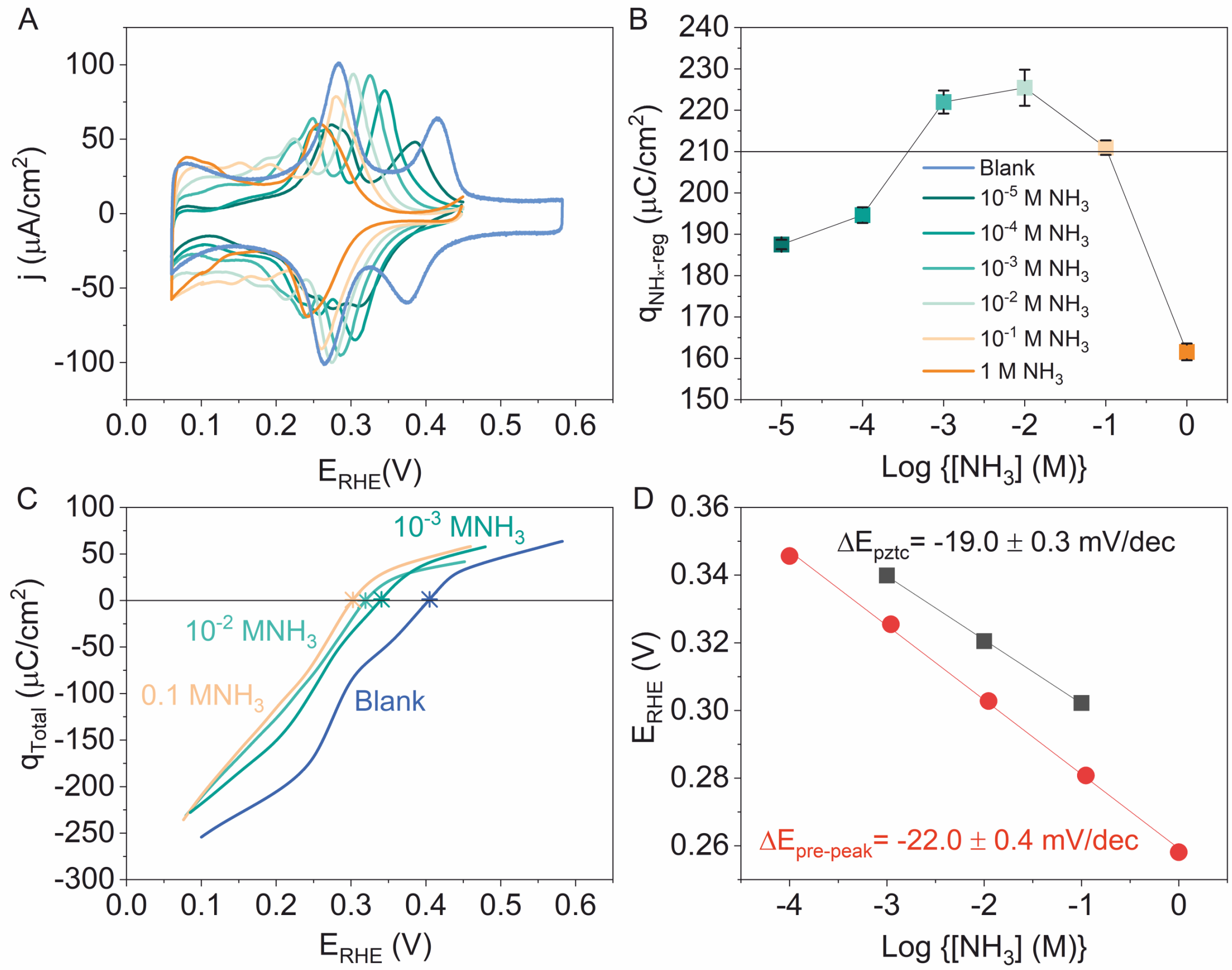

Supplementary Figure 7. Analysis of charge evolution in the H^+/OH^- adsorption region on Pt. (A) Voltammetric profiles of polycrystalline Pt at different ammonia concentrations. The potential window shown corresponds to the region where hydrogen and hydroxyl adsorption–desorption processes take place. **(B)** Evolution of the integrated charge derived from the voltammograms in panel A as a function of log[NH_3]. **(C)** Variation of the total charge with potential for the blank and for ammonia concentrations of 10^{-3} M, 10^{-2} M, and 0.1 M. The crossing points indicate the potentials of zero total charge (pztc). **(D)** Comparison between the variation of the pztc and the potential of the pre-peak (associated with the Pt(100) facet) as a function of log[NH_3]. Scan rate: 50mV/s; 0.1M KOH.

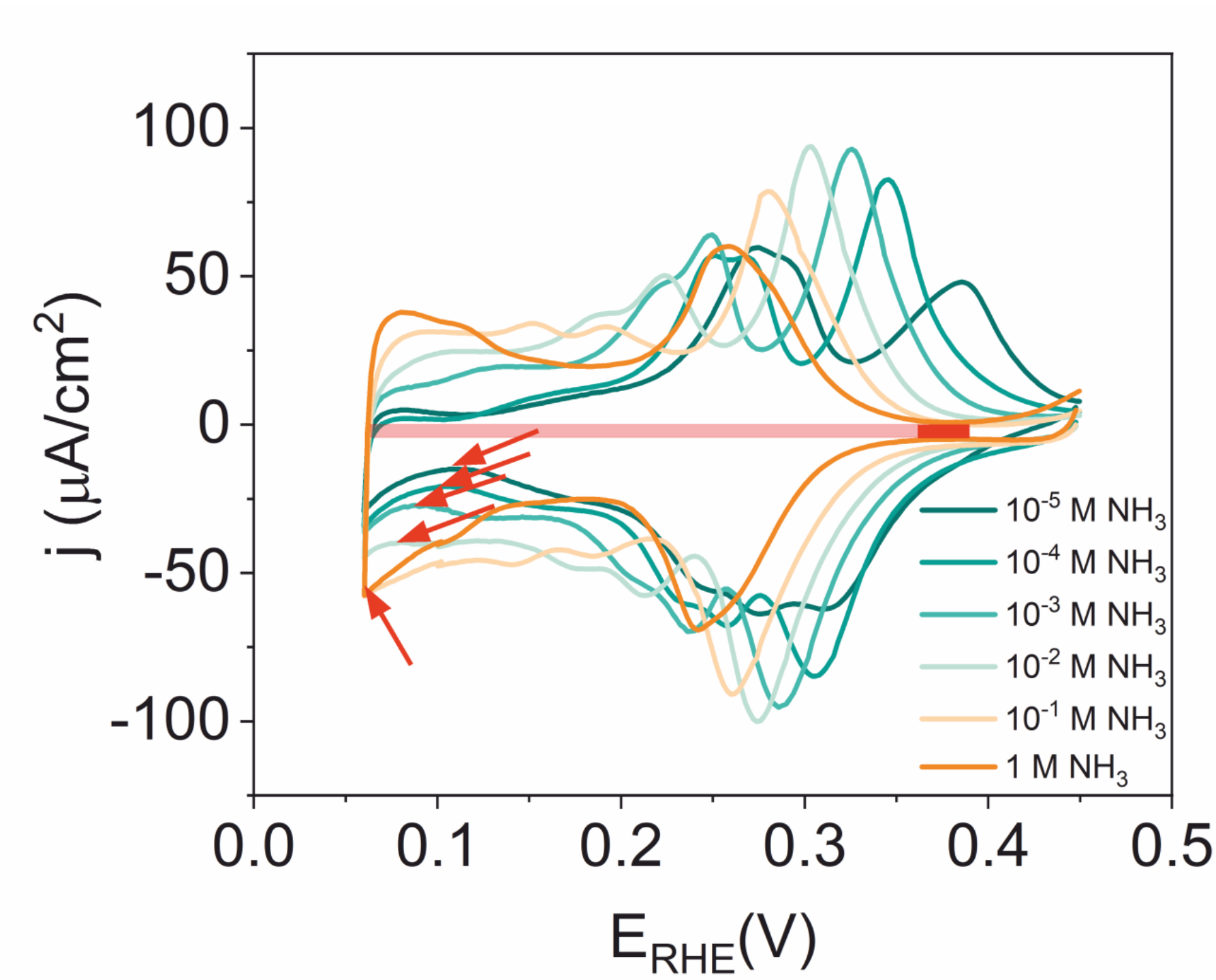

Supplementary Figure 8. Schematic for the criterion used to determine the integrated charge from cyclic voltammograms. Red arrows indicate the lower potential limit used for integration at every ammonia concentration. Scan rate: 50mV/s; 0.1M KOH.

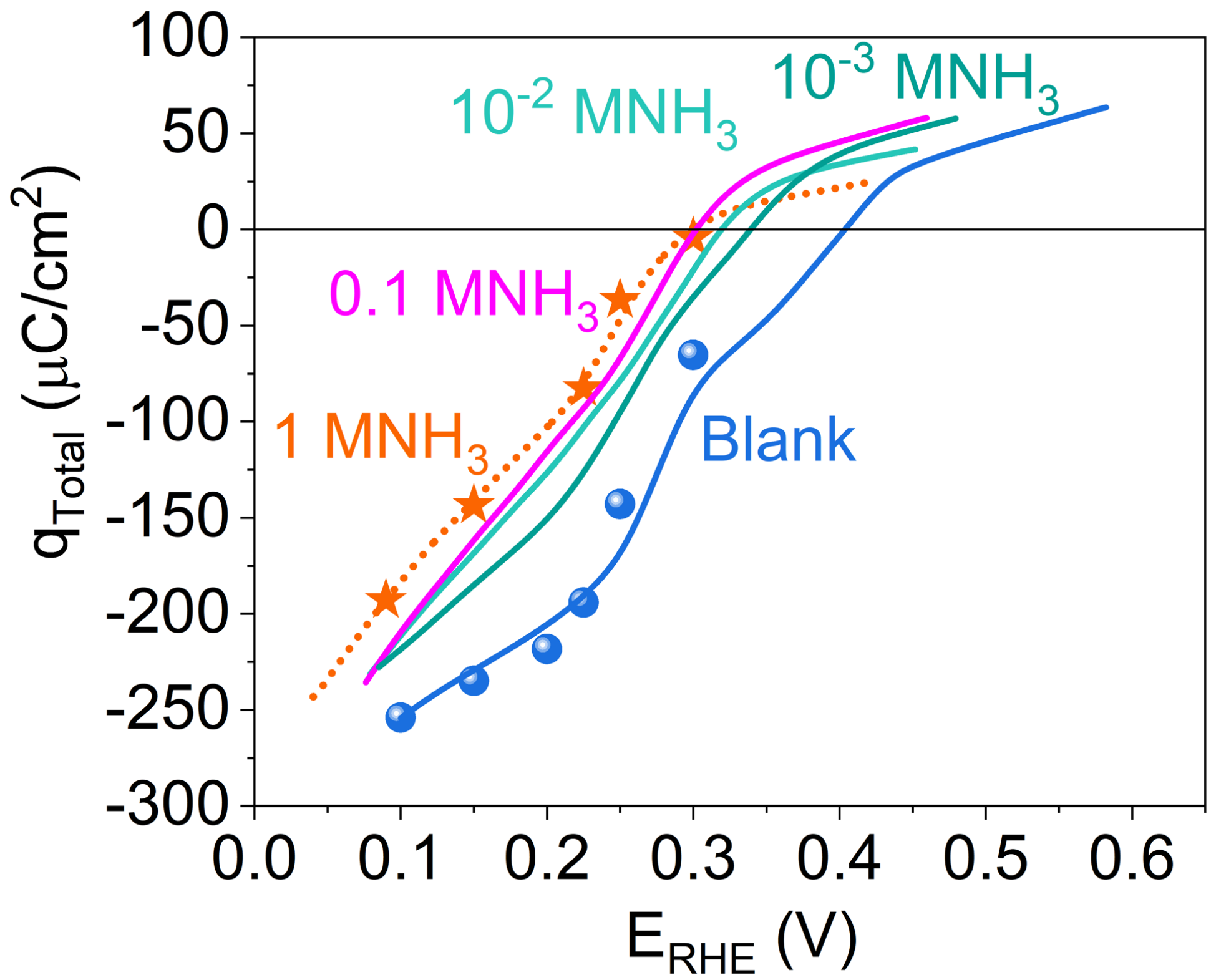

Supplementary Figure 9. Determination of the potential of zero total charge (pztc). Plot of the variation of total charge with potential for Pt(poly) in 0.1M KOH without ammonia containing solution and for 10^{-3} M, 10^{-2} M, 0.1 M and 1 M NH_3.

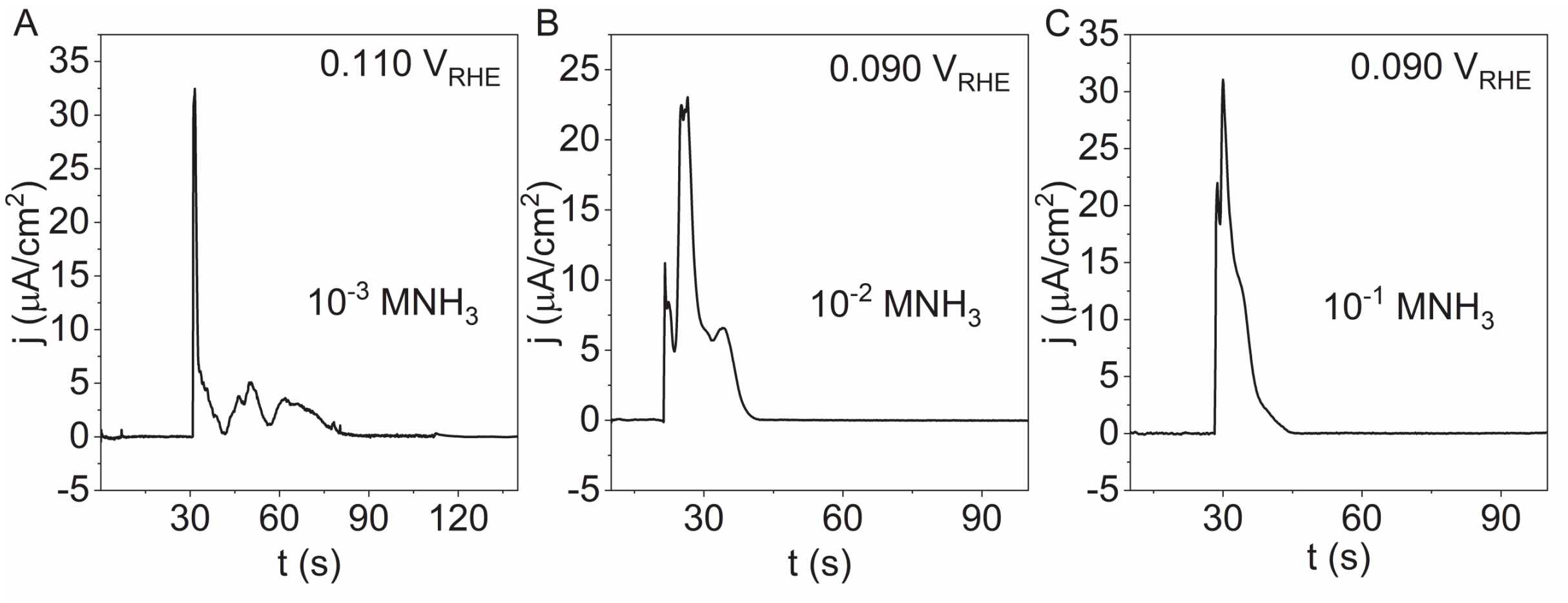

Supplementary Figure 10. CO-displacement current signals. The transient currents were obtained from CO charge-displacement experiments for ammonia concentrations of 10^{-3} M, 10^{-2} M, and 10^{-1} M at their corresponding potentials.

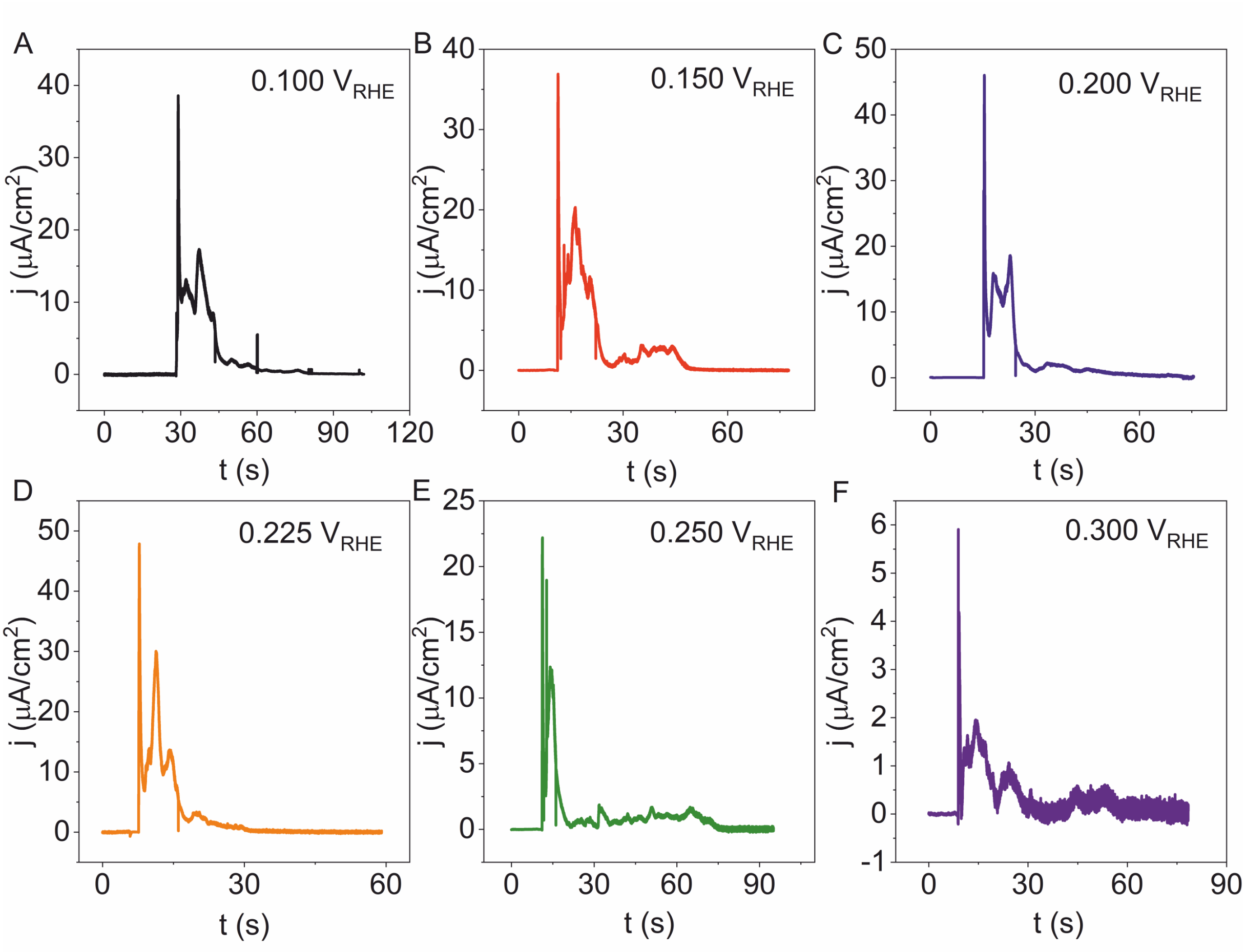

Supplementary Figure 11. CO-displacement current signals. The transient currents were obtained from CO charge-displacement experiments at different potentials in the absence of ammonia. The charge associated with the current recorded at 0.1V_{RHE} was used as the integration constant.

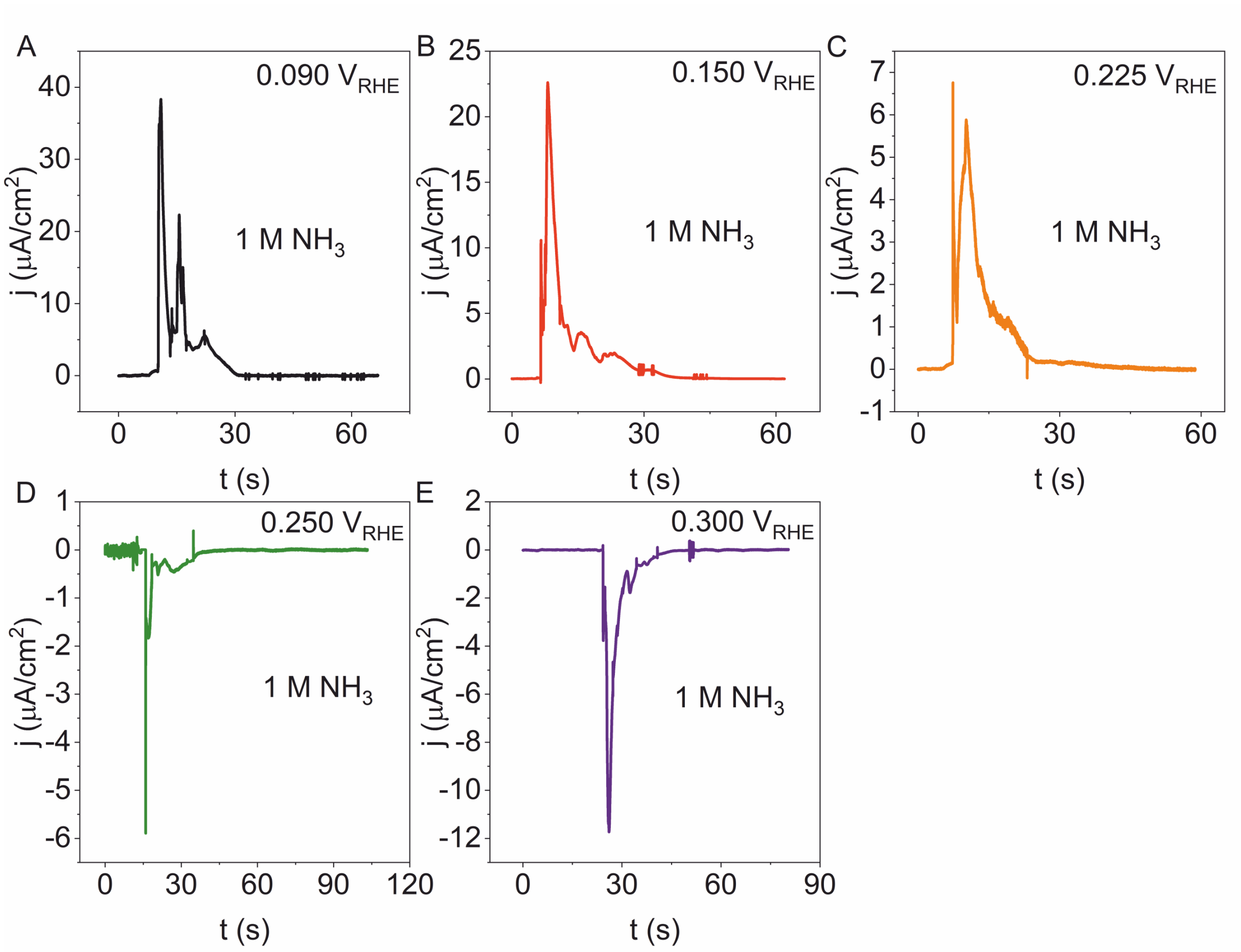

Supplementary Figure 12. CO-displacement current signals. The transient currents were obtained from CO charge-displacement experiments at different potentials in the presence of 1 M NH_3. The charge associated with the current recorded at 0.09V_{RHE} was used as the integration constant. The difference in charge between 0.225 and 0.250 clearly shows a greater contribution of OH^- species to the displaced charge.

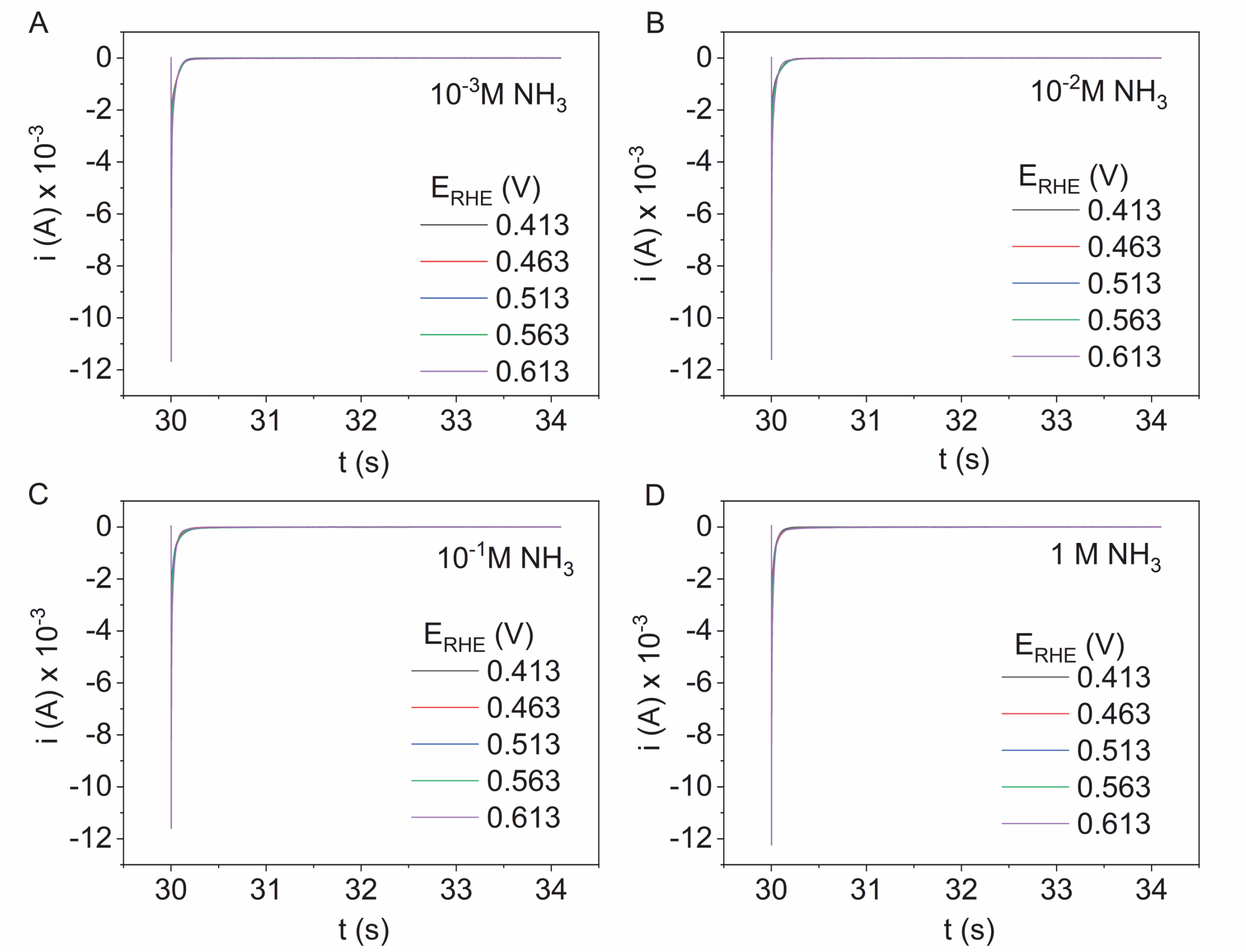

Supplementary Figure 13. Stripping of the adsorbed species during ammonia oxidation. Currents recorded at 0.1 V_{RHE} after potential steps from 0.413 V, 0.463 V, 0.513 V, 0.563 V, and 0.613 V for 10^{-3} M, 10^{-2} M, 10^{-1} M, and 1 M NH_3.

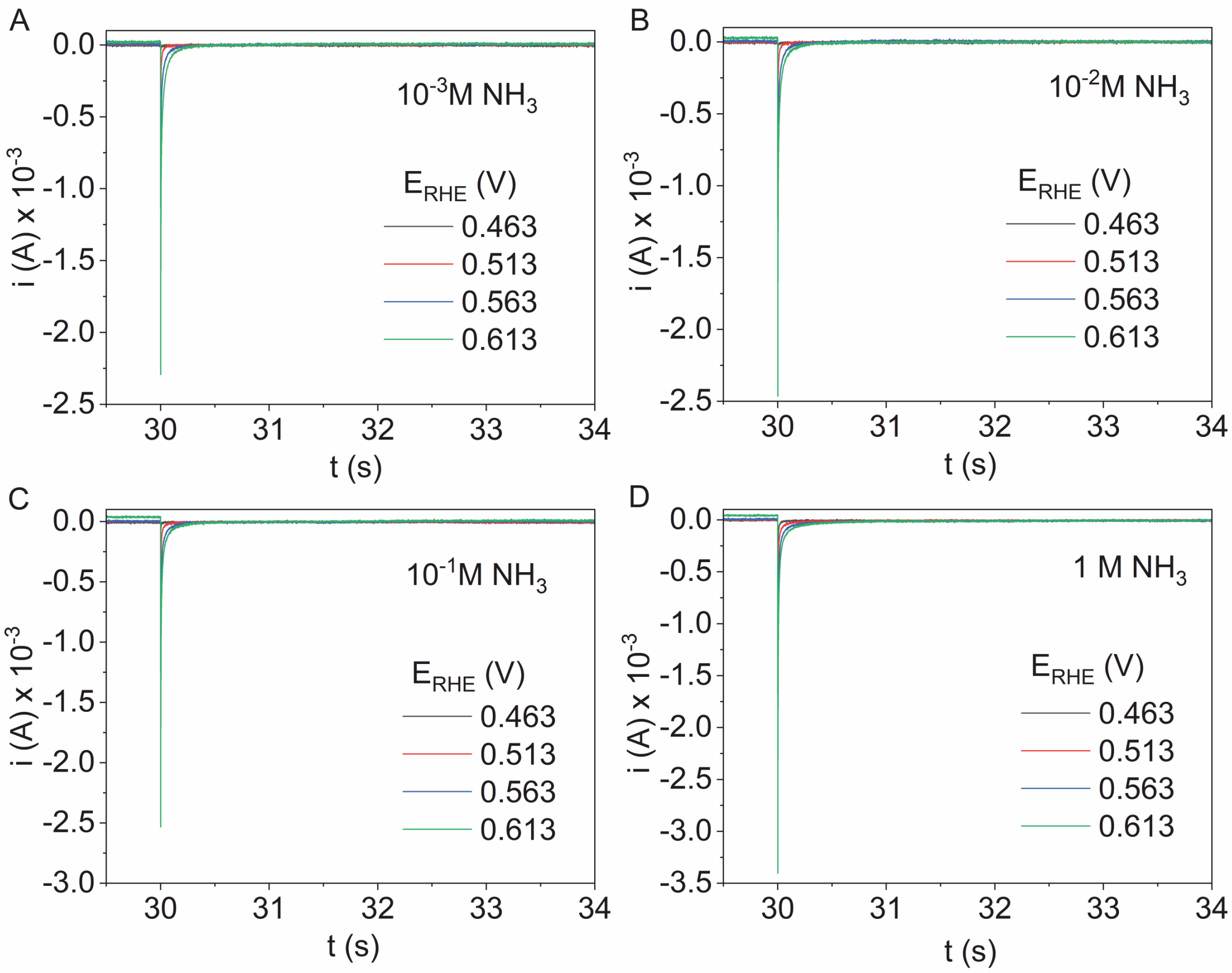

Supplementary Figure 14. Potential jumps within the N_2 evolution region during AOR used to the subtraction in stripping currents. Currents recorded at 0.413 V_{RHE} after potential steps from 0.463 V, 0.513 V, 0.563 V, and 0.613 V for 10^{-3} M, 10^{-2} M, 10^{-1} M, and 1 M NH_3.

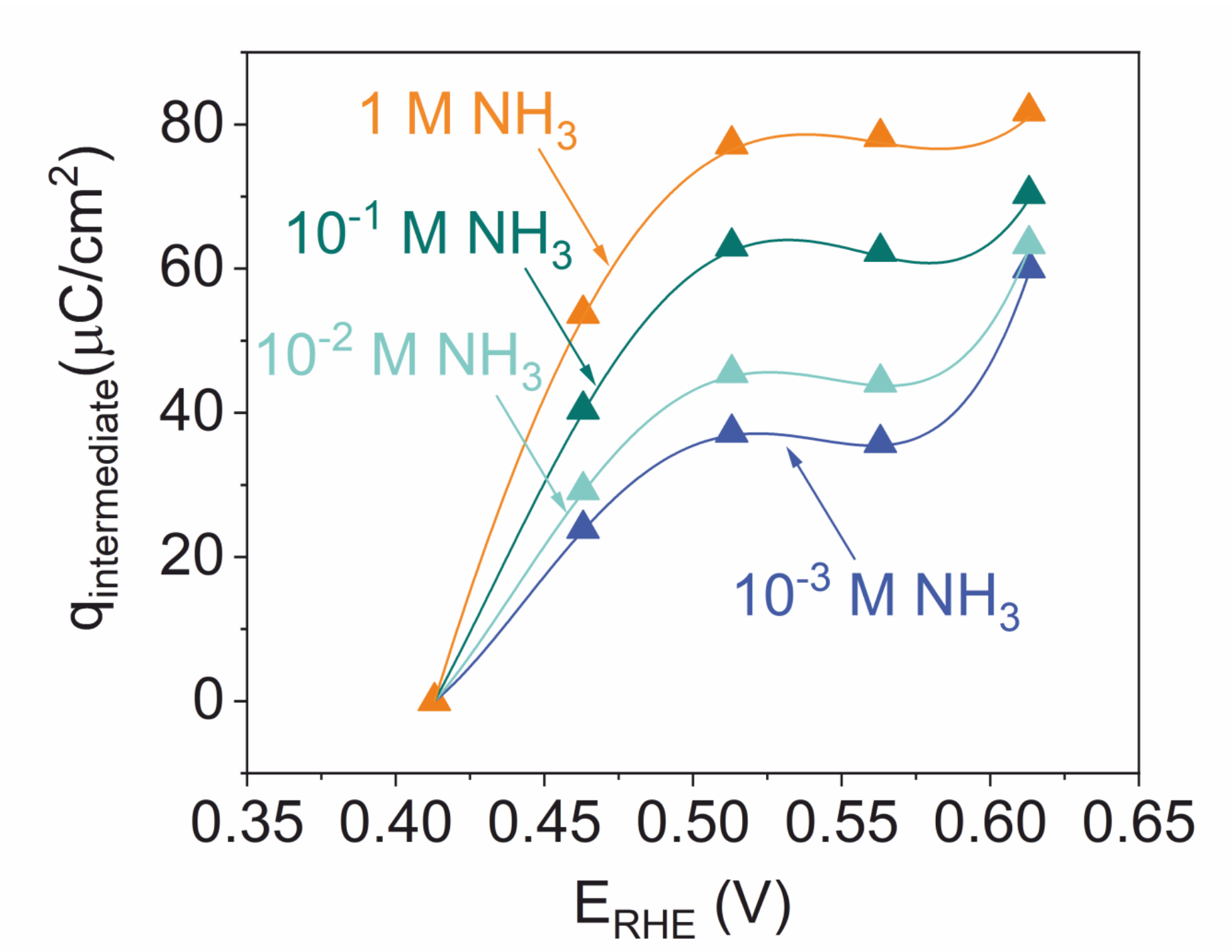

Supplementary Figure 15. Plot of $q_{intermediates}$ vs. potential. Increasing ammonia concentration enhances the charge associated with reaction intermediates.

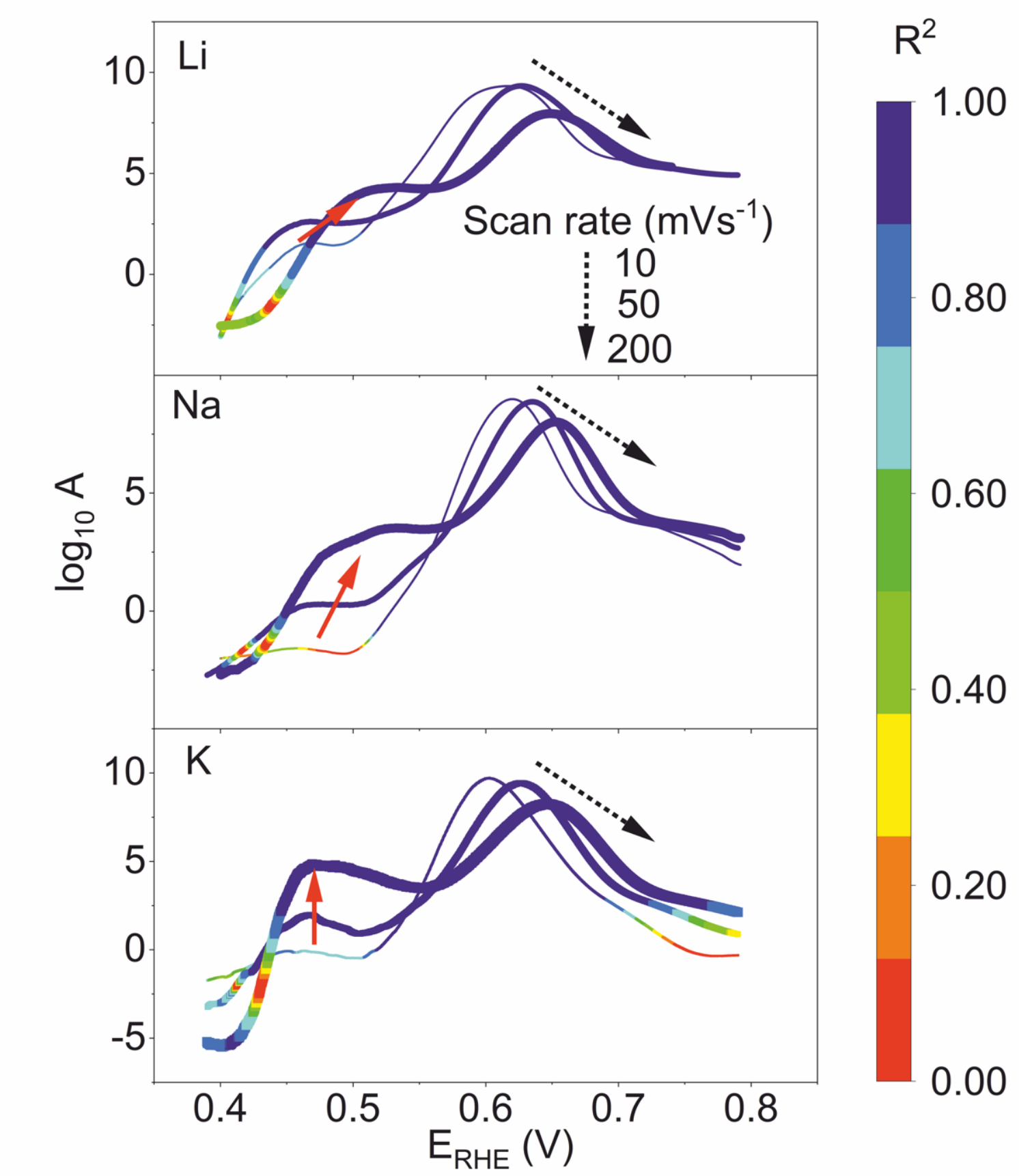

Supplementary Figure 16. **Relationship between water structure and Arrhenius pre-exponential factor.** Bias and scan-rate dependence of $\log_{10}$ A for different alkali-metal cations (Li^+, Na^+ and K^+).

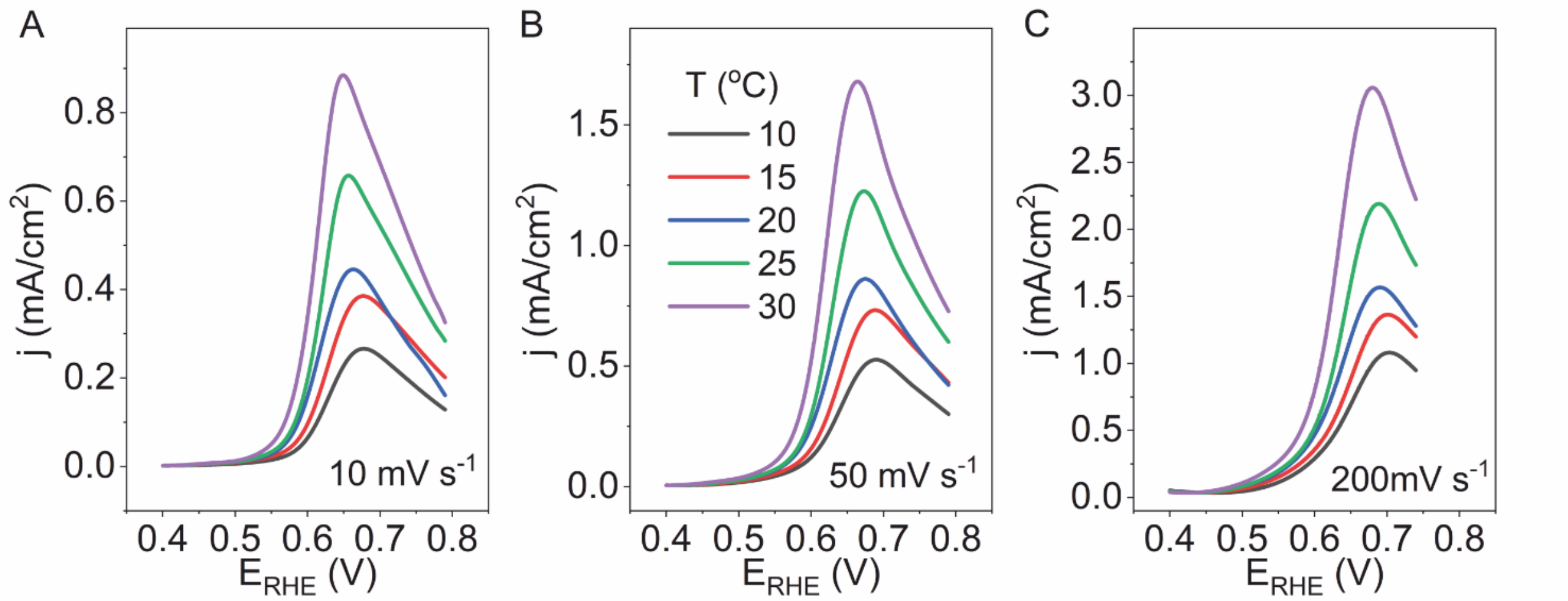

Supplementary Figure 17. Positive going scan of the CVs in the N_2 evolution region during AOR. Current densities used to extract the kinetic parameters shown in Supplementary Figure 16 and Figure 5C of the main text. 0.1M LiOH + 0.1M NH_3.

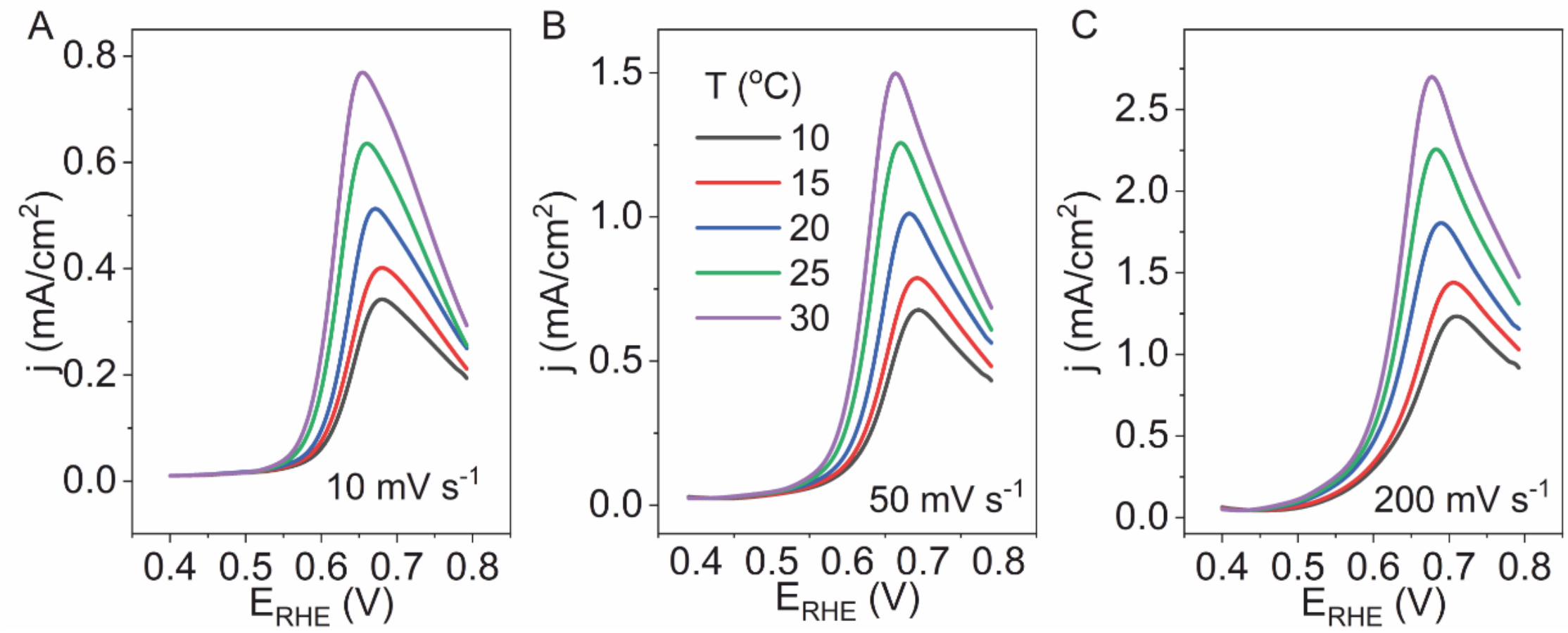

Supplementary Figure 18. Positive going scan of the CVs in the N_2 evolution region during AOR. Current densities used to extract the kinetic parameters shown in Supplementary Figure 16 and Figure 5C of the main text. 0.1M NaOH + 0.1M NH_3.

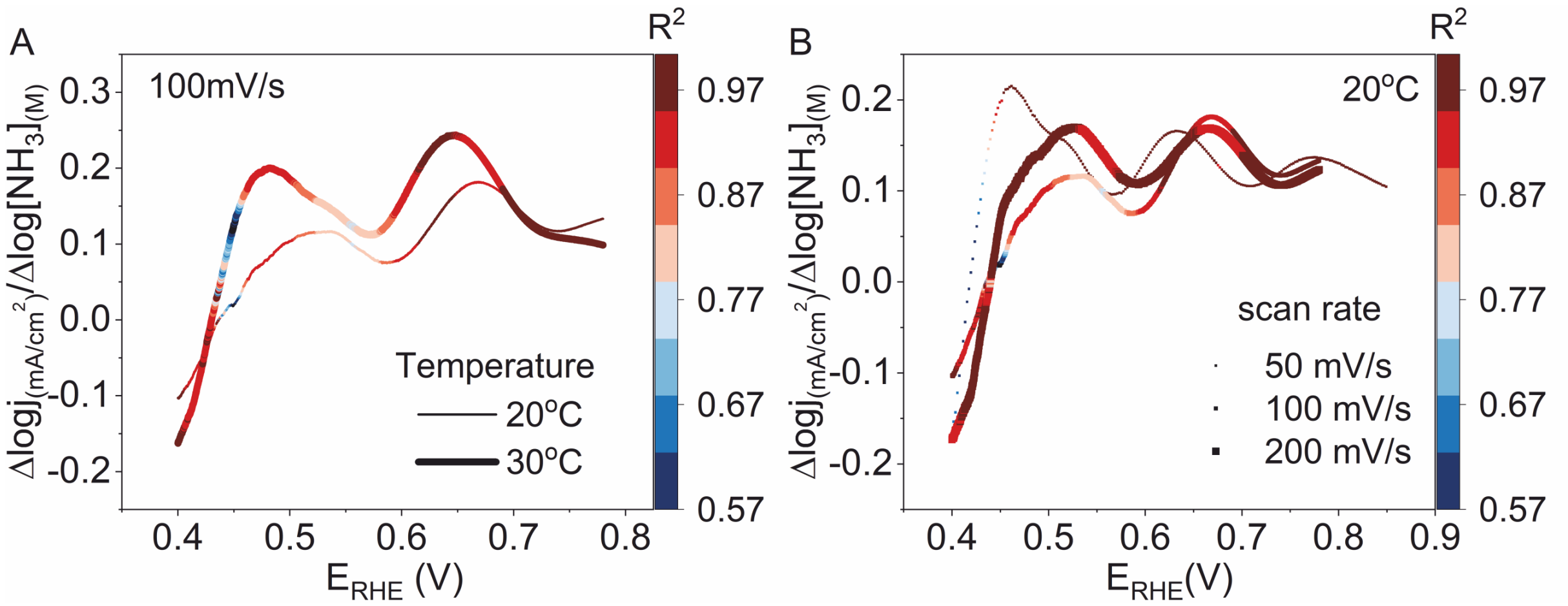

Supplementary Figure 19. **Potential-dependent evolution of the reaction order. (A)** Plot of logj/log[NH_3] versus potential at 20°C and 30°C for a scan rate of 100 mV s^{-1}. **(B)** Plot of logj/log[NH_3] versus potential at 20°C for scan rates of 50, 100 and 200 mV s^{-1}.

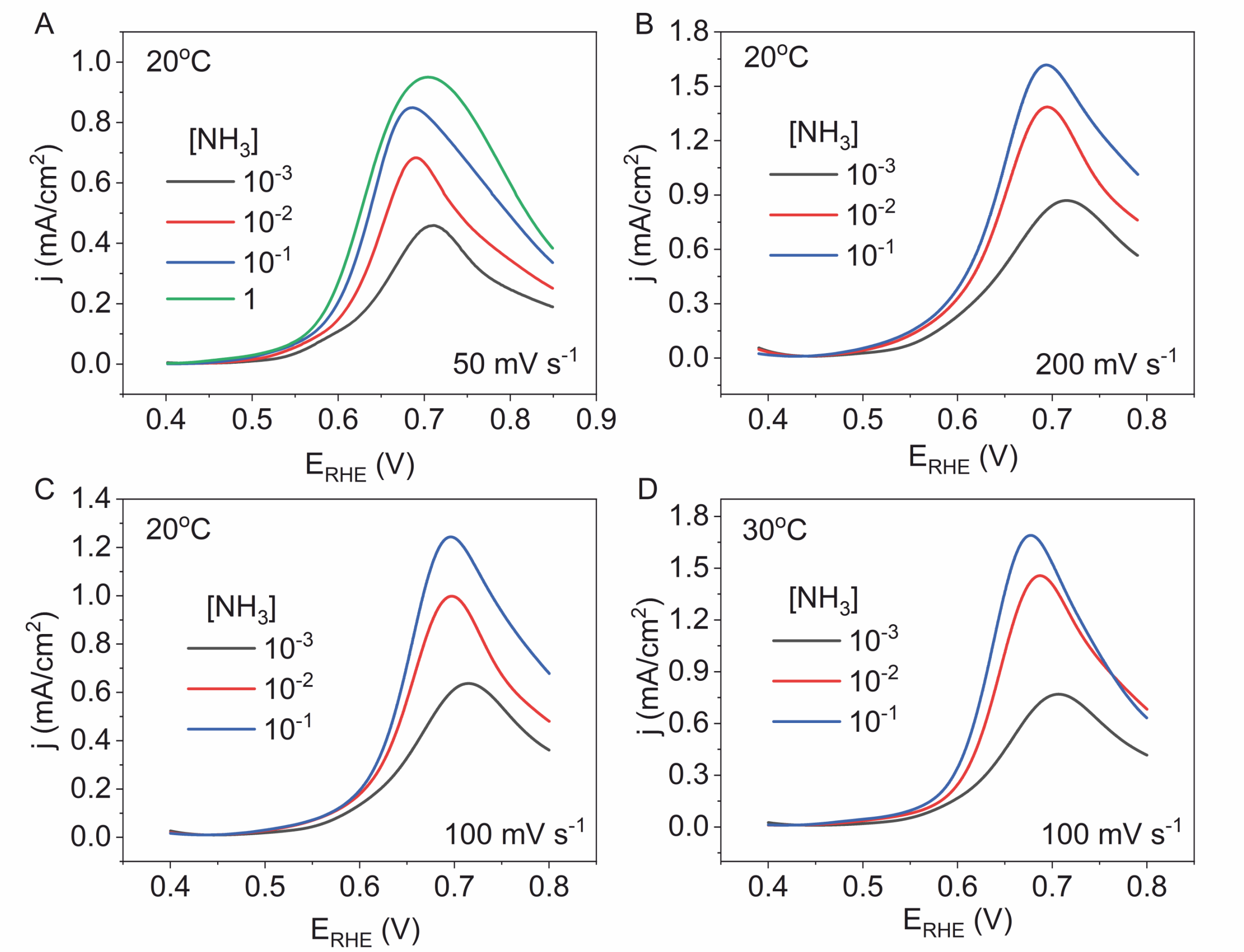

Supplementary Figure 20. Positive going scan of the CVs in the N_2 evolution region during AOR. Current density as a function of potential for the different ammonia concentrations used in the reaction order calculations at 20°C and 30 °C, recorded at scan rates of 50, 100, and 200 mV s^{-1}.

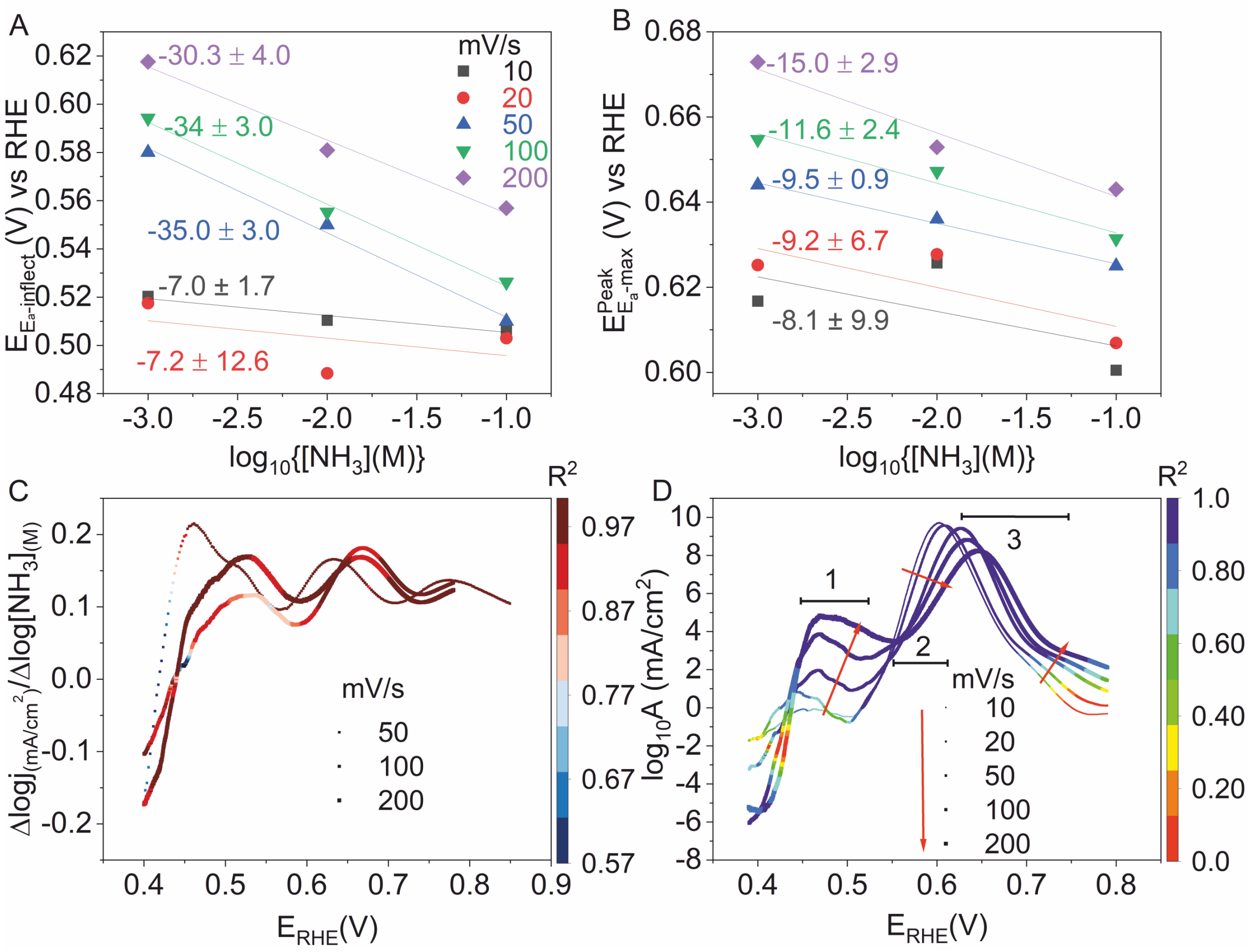

Supplementary Figure 21. **Identification of kinetic differences in parallel processes via kinetic parameters.** (**A**) Potentials at the E_a inflection point vs log[NH_3] for the different scan rates. (**B**) Potentials at maximum E_a vs log[NH_3] for the same scan rates. Note, for the lowest scan rates, the currents are the smallest and, thus, the uncertainty is larger. (**C**) Slope of log j/log[NH_3] (from plots of NH_3 oxidation current vs log[NH_3] at 20 °C; see Supplementary Note 5) vs E at 50, 100, and 200 mV s^{-1}. (**D**) Logarithm of the pre-exponential factor (A) vs E for different scan rates in 0.1M NH_3. Experimental conditions: 0.1M KOH; scan rates: 10, 20, 50, 100 and 200 mVs^{-1}. Ammonia concentrations: 10^{-3}, 10^{-2} and 10^{-1} M NH_3. Temperatures used in the study: 10, 15, 20, 25 and 30 °C.

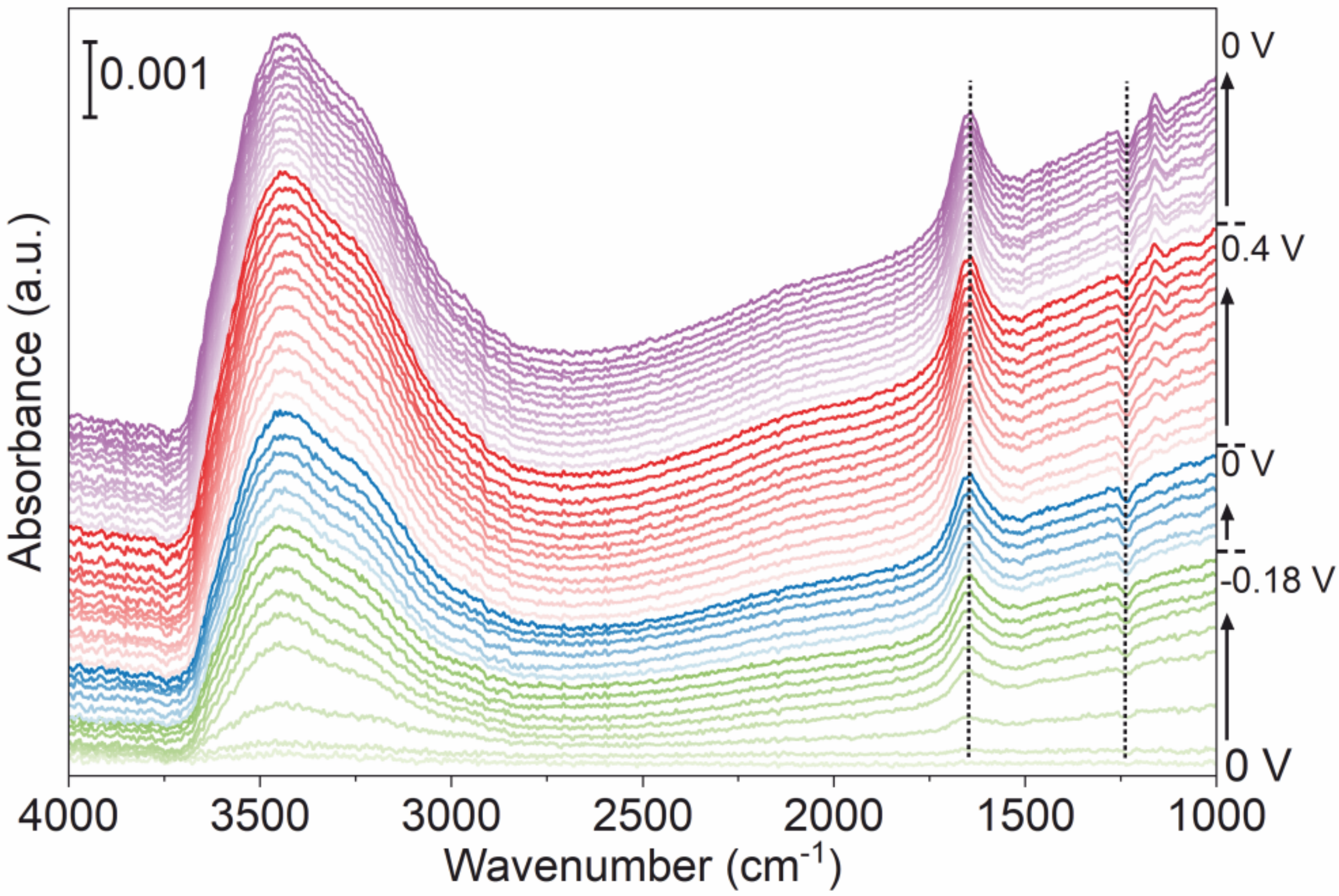

Supplementary Figure 22. ATR-SEIRAS spectra for the polycrystalline Pt surface in absence of NH_3. Evolution of the interfacial spectral features during the positive- and negative-going scans recorded at 5 mVs^{-1} in 0.1M KOH.

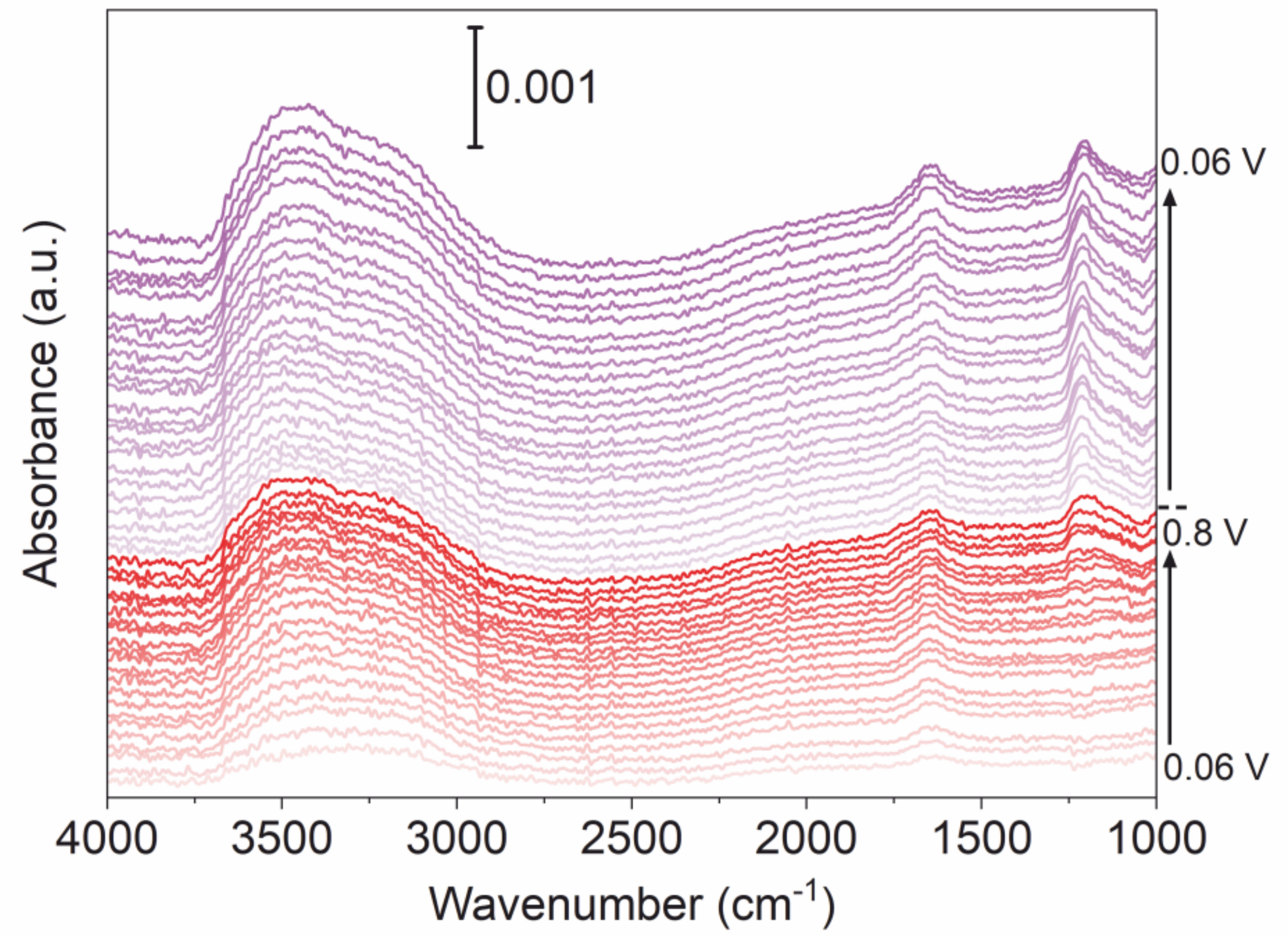

Supplementary Figure 23. ATR-SEIRAS spectra for the polycrystalline Pt surface under ammonia oxidation reaction (AOR) conditions. Evolution of the interfacial spectral features during the positive- and negative-going scans recorded at 5 mVs^{-1} in 0.1M KOH containing 0.1M NH_3.

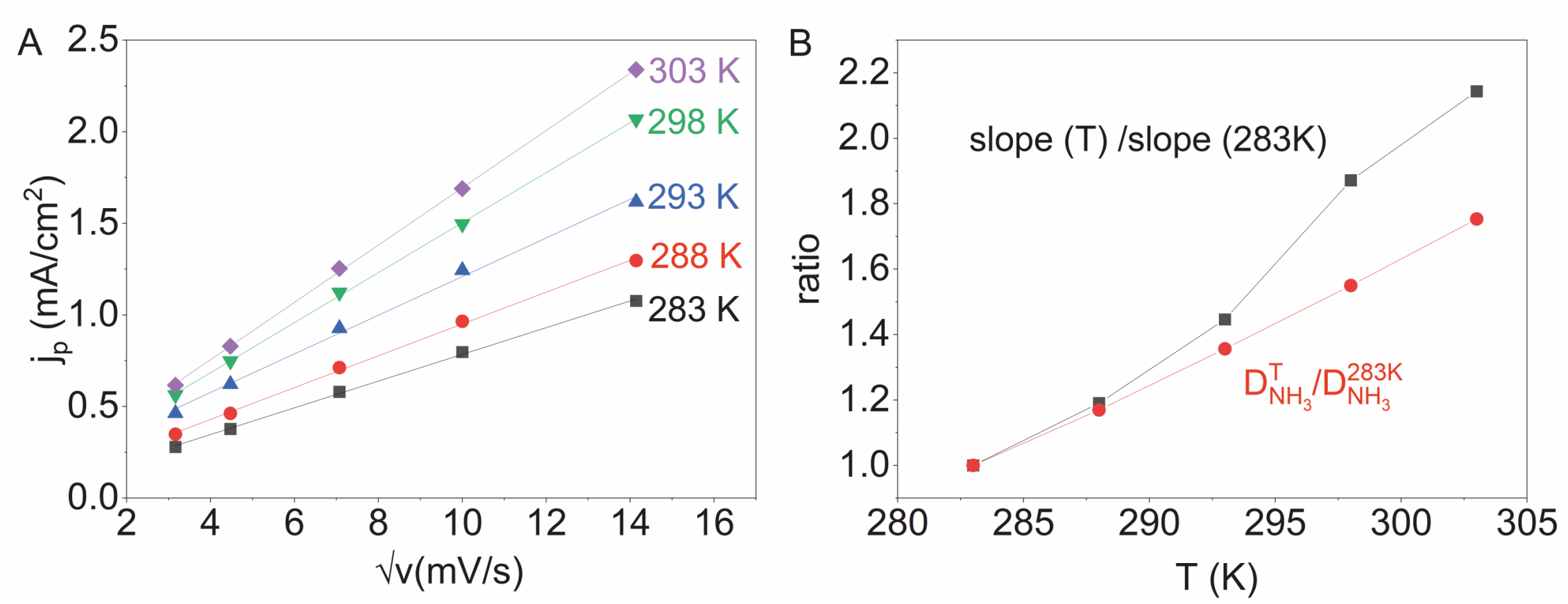

Supplementary Figure 24. **Analysis of the variation of peak current density with scan rate and temperature. (A)** Plot of the peak current density vs of the square root of the scan rate at the different temperatures used in this study. **(B)** Ratio of the slopes $j_p/\sqrt{v}$, normalized to the slope at the lowest temperature, and ratio of the ammonia diffusion coefficient normalized to its value at the lowest temperature, both plotted as a function of temperature.

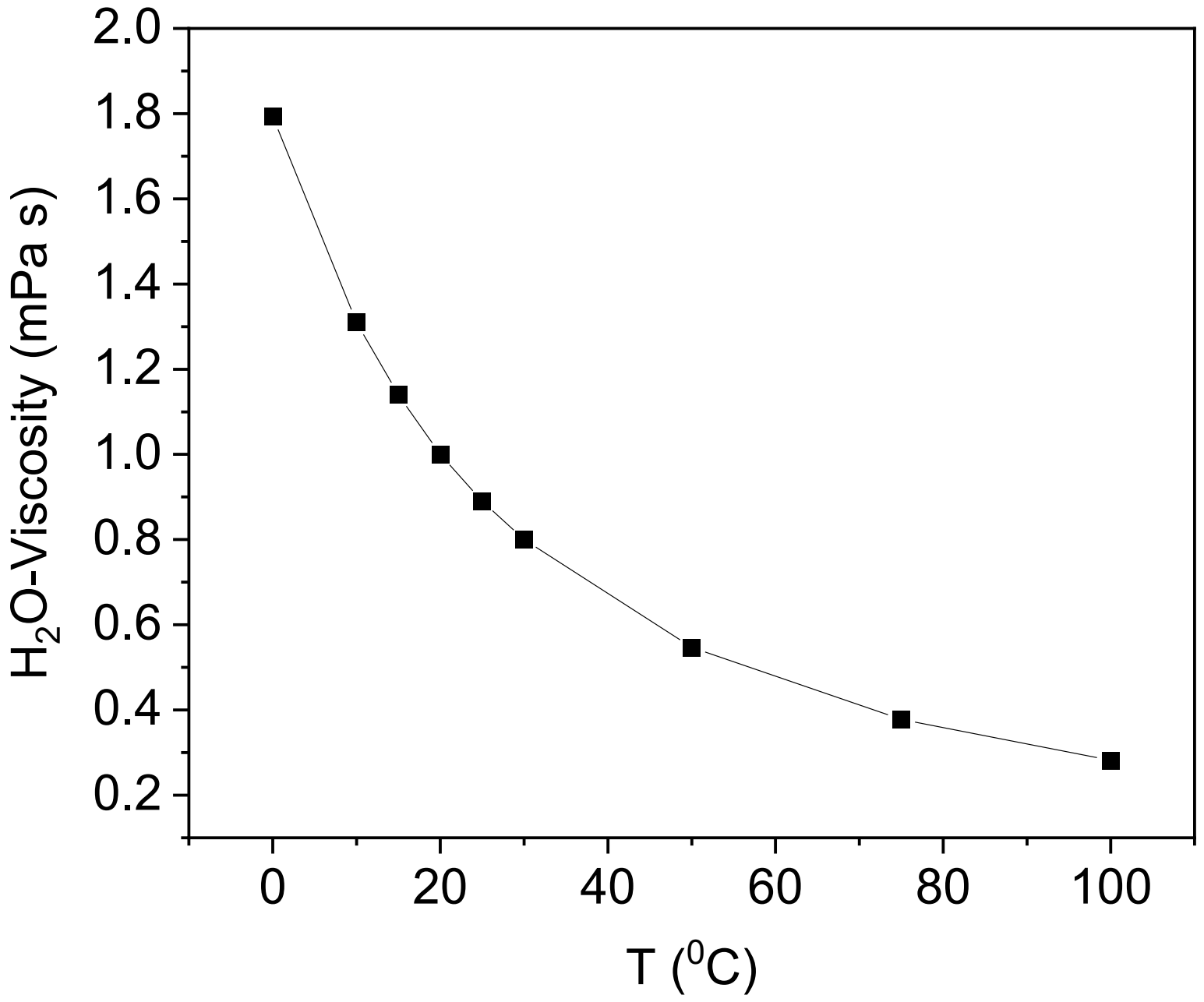

Supplementary Figure 25. Temperature dependence of water viscosity. Data are based on interpolation of literature values[20].

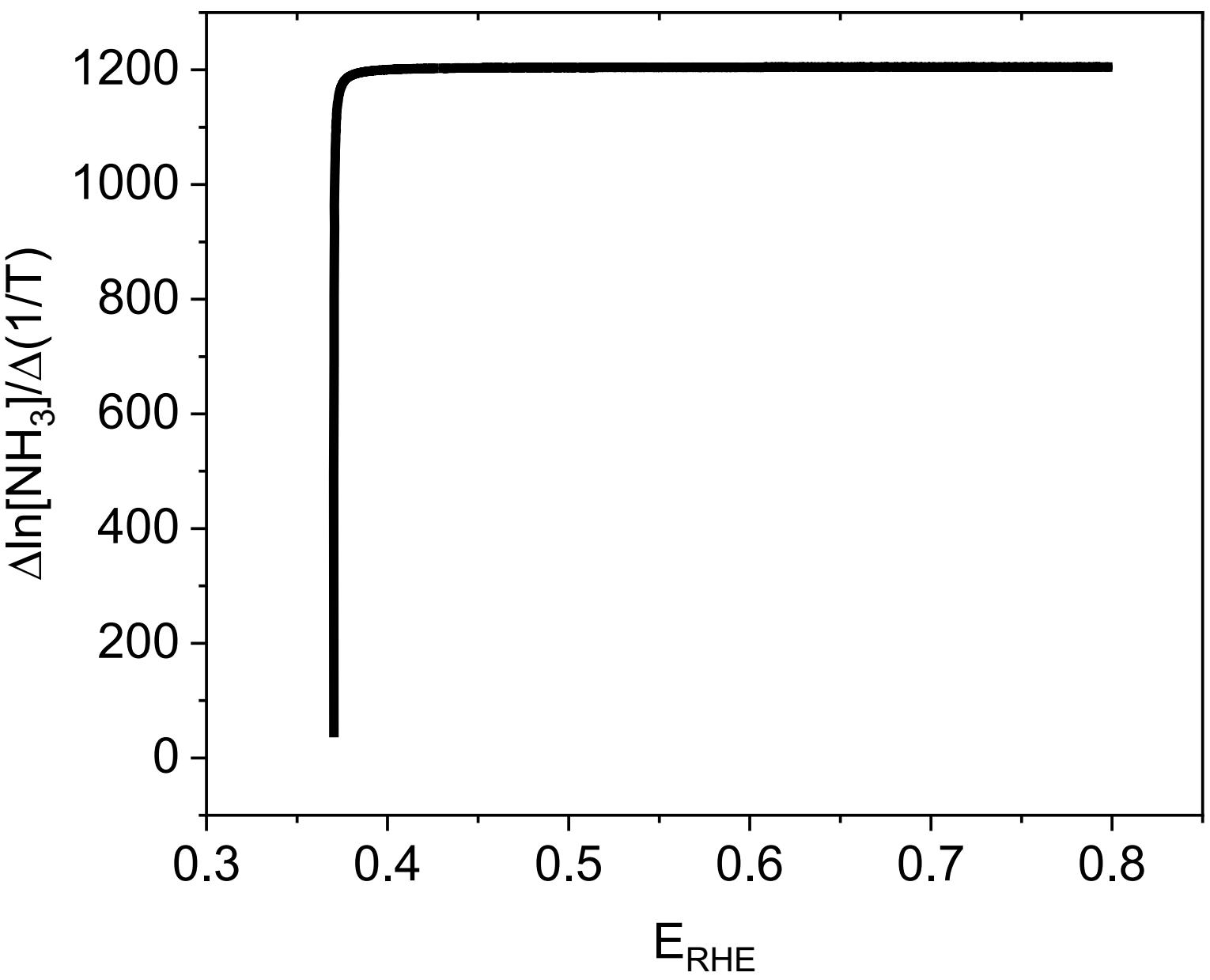

Supplementary Figure 26. Variation of the contribution to the chemical potential vs potential. The values correspond to the distance between the outer Helmholtz plane and the electrode surface (~1 nm).

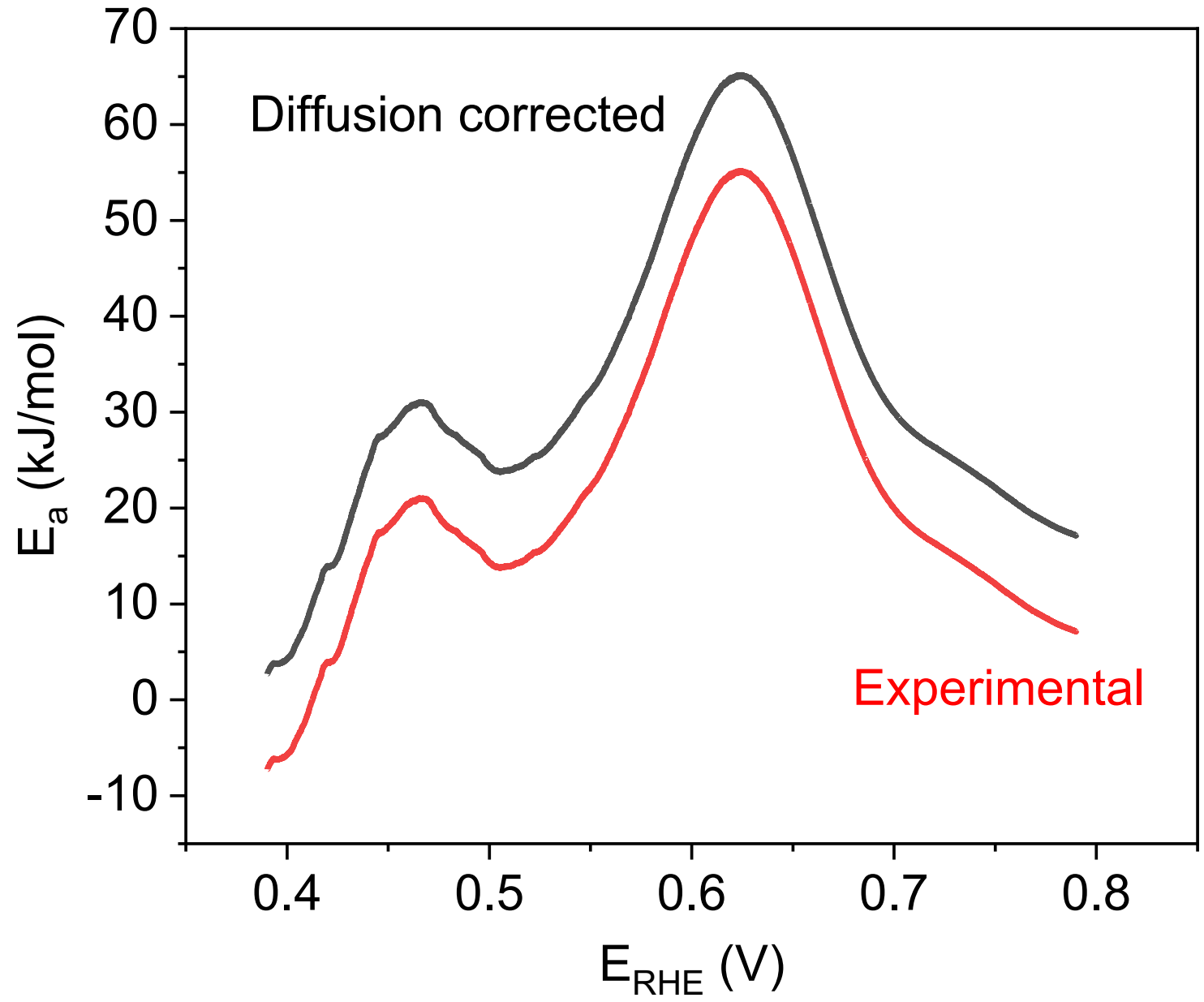

Supplementary Figure 27. Impact of diffusion on the activation energy. Experimental result (red) for 0.1M NH_3 in 0.1M KOH and after diffusion correction (black) for a distance of 1 nm with respect to the electrode surface (outer Helmholtz plane).

$[NH_3]$	E_{RHE}^{dis} (V)	q_{dis} ($\mu C/cm^2$)
10^{-3}M	0.100	160
10^{-2}M	0.090	150
10^{-1}M	0.090	147

Supplementary Table 1. Charge values obtained from the transients recorded during the CO charge-displacement experiments, used as integration constants for ammonia concentrations of 10^{-3} M, 10^{-2} M, and 10^{-1} M.

Blank			
E/V (RHE)	q_{dis} (μC/cm2)	C(μF/cm2)	σ(μC/cm2)
0.1	204	30	-254
0.15	186	30	-235
0.2	171	30	-218
0.225	148	30	-194
0.25	97	30	-143
0.3	21	30	-65

Supplementary Table 2. Charge values associated with the transient currents shown in Supplementary Figure 9.

1M NH3			
E/V (RHE)	q_{dis} (μC/cm2)	C(μF/cm2)	q(μC/cm2)
0.09	143	30	-193
0.15	95	30	-143
0.225	36	30	-82
0.25	-9	30	-36
0.3	-40	30	-4

Supplementary Table 3. Charge values associated with the transient currents shown in Supplementary Figure 9.

E/V (RHE)	0.001M	0.01M	0.1M	1M
0.413	233	254	235	190
0.463	257	283	275	251
0.513	279	312	318	298
0.563	318	352	353	342
0.613	405	400	382	406

Supplementary Table 4. $q_{stripping}$ values (μC/cm^2) obtained by integrating the currents shown in Supplementary Figure 13.

E/V (RHE)	0.001M	0.01M	0.1M	1M
0.413	0	0	0	0
0.463	0	0	0	7
0.513	8	13	20	31
0.563	50	54	56	74
0.613	112	83	77	134

Supplementary Table S5. $q_{E_{AOR}-0.413V}$ values (μC/cm^2) obtained by integrating the currents shown in Figure Supplementary Figure 14.

E/V (RHE)	0.001M	0.01M	0.1M	1M
0.413	0	0	0	0
0.463	24	29	40	61
0.513	46	58	83	108
0.563	85	98	118	152
0.613	172	146	147	216

Supplementary Table 6. Resulting charge values after subtracting the contribution associated with the hydrogen and hydroxyl adsorption region recorded between 0.4 and 0.1 V.

E/V (RHE)	0.001M	0.01M	0.1M	1M
0.413	0	0	0	0
0.463	24	29	40	54
0.513	38	42	63	77
0.563	35	44	62	78
0.613	60	63	70	82

Supplementary Table 7. $q_{intermediates}$ values obtained after subtracting the charge contributions associated with the hydrogen and hydroxyl adsorption region (recorded between 0.4 and 0.1 V) and $q_{E_{AOR}-0.413V}$.

				$[\Delta E_{Alk}^{RHE}(T) - \Delta E_{Alk}^{RHE}(25°C)]\ (V)$		
T (°C)	$[N_2]$ M	$k_H^{N_2}$(mol l^{-1}atm^{-1})	$k_H^{NH_3}$(mol l^{-1}atm^{-1})	0.1M NH_3	0.01 NH_3	0.001 NH_3
10	0.000848	0.000846	126.1	-0.00163	-0.00262	-0.00361
15	0.000785	0.000767	97.5	-0.00094	-0.00160	-0.00226
20	0.000669	0.000703	76.0	-0.00064	-0.00097	-0.00130
25	0.000625	0.000652	59.8	0.00000	0.00000	0.00000
30	0.000580	0.000610	47.4	0.000572	0.00090	-0.00219

Supplementary Table 8. Nitrogen solubility[20] and Henry's law constants[21] for nitrogen and ammonia in water at each temperature investigated, as obtained from the literature. The right-hand columns show the change in equilibrium potential shift relative to 25 °C for each ammonia concentration and temperature.

Supplementary References

(1) Mao, Z.; Campbell, C. T. Apparent Activation Energies in Complex Reaction Mechanisms: A Simple Relationship via Degrees of Rate Control. *ACS Catal.* **2019**, *9* (10), 9465–9473. https://doi.org/10.1021/acscatal.9b02761.

(2) Stegelmann, C.; Andreasen, A.; Campbell, C. T. Degree of Rate Control: How Much the Energies of Intermediates and Transition States Control Rates. *J. Am. Chem. Soc.* **2009**, *131* (23), 8077–8082. https://doi.org/10.1021/ja9000097.

(3) Calderón-Cárdenas, A.; Paredes-Salazar, E. A.; Varela, H. Apparent Activation Energy in Electrochemical Multistep Reactions: A Description via Sensitivities and Degrees of Rate Control. *ACS Catal.* **2020**, *10* (16), 9336–9345. https://doi.org/10.1021/acscatal.0c02359.

(4) Lizée, M.; Silva Olaya, A. R.; Druce, J.; Roldan Cuenya, B.; Oener, S. Z. Disentangling Multistep Kinetics by Combining Electrochemical Arrhenius Analysis with Micro-Kinetic Modelling. *Archive* **2025**.

(5) Rodellar, C. G.; Gisbert-Gonzalez, J. M.; Sarabia, F.; Roldan Cuenya, B.; Oener, S. Z. Ion Solvation Kinetics in Bipolar Membranes and at Electrolyte–Metal Interfaces. *Nat. Energy* **2024**, *9* (5), 548–558. https://doi.org/10.1038/s41560-024-01484-z.

(6) Gisbert-González, J. M.; Rodellar, C. G.; Druce, J.; Ortega, E.; Cuenya, B. R.; Oener, S. Z. Bias Dependence of the Transition State of the Hydrogen Evolution Reaction. *J. Am. Chem. Soc.* **2025**. https://doi.org/10.1021/jacs.4c18638.

(7) Silva Olaya, A. R.; Druce, J.; Gisbert-Gonzalez, J. M.; Roldan Cuenya, B.; Oener, S. Z. Pressure and Bias Dependence of the Rate-Limiting Steps of the Oxygen Reduction Reaction. *Nat. Commun.* **2025**, *Accepted*.

(8) Sarabia, F.; Gomez Rodellar, C.; Roldan Cuenya, B.; Oener, S. Z. Exploring Dynamic Solvation Kinetics at Electrocatalyst Surfaces. *Nat. Commun.* **2024**, *15* (1), 8204. https://doi.org/10.1038/s41467-024-52499-9.

(9) Martínez-Hincapié, R.; Timoshenko, J.; Wagner, T.; Ortega, E.; Druce, J.; Monteiro, M. C. O.; Rüscher, M.; Jang, J.; Alagöz, E. Ö.; Lasagna, S.; Jacobse, L.; Bergmann, A.; Cuenya, B. R.; Oener, S. Z. Interfacial Solvation Pre-Organizes the Transition State of the Oxygen Evolution Reaction. *Nat. Chem.* **2025**, 1–9. https://doi.org/10.1038/s41557-025-01932-7.

(10) Tian, X.; Tosello Gardini, A.; Raucci, U.; Xiao, H.; Zhuo, Y.; Parrinello, M. Electrochemical Potential-Driven Water Dynamics Control CO2 Electroreduction at the Ag/H2O Interface. *Nat. Commun.* **2025**, *16* (1), 10636. https://doi.org/10.1038/s41467-025-65630-1.

(11) Wang, H.; Dekel, D. R.; Abruña, H. D. Unraveling the Mechanism of Ammonia Electrooxidation by Coupled Differential Electrochemical Mass Spectrometry and Surface-Enhanced Infrared Absorption Spectroscopic Studies. *J. Am. Chem. Soc.* **2024**, *146* (23), 15926–15940. https://doi.org/10.1021/jacs.4c02621.

(12) Katsounaros, I.; Chen, T.; Gewirth, A. A.; Markovic, N. M.; Koper, M. T. M. Evidence for Decoupled Electron and Proton Transfer in the Electrochemical Oxidation of Ammonia on Pt(100). *J. Phys. Chem. Lett.* **2016**, *7* (3), 387–392. https://doi.org/10.1021/acs.jpclett.5b02556.

(13) Rizo, R.; Sitta, E.; Herrero, E.; Climent, V.; Feliu, J. M. Towards the Understanding of the Interfacial pH Scale at Pt(1 1 1) Electrodes. *Electrochimica Acta* **2015**, *162*, 138–145. https://doi.org/10.1016/j.electacta.2015.01.069.

(14) Weaver, M. J.; Chang, S.-C.; Leung, L.-W. H.; Jiang, X.; Rubel, M.; Szklarczyk, M.; Zurawski, D.; Wieckowski, A. Evaluation of Absolute Saturation Coverages of Carbon Monoxide on Ordered Low-Index Platinum and Rhodium Electrodes. *J. Electroanal. Chem.* **1992**, *327* (1–2), 247–260. https://doi.org/10.1016/0022-0728(92)80151-S.

(15) Cuesta, A. Measurement of the Surface Charge Density of CO-Saturated Pt(111) Electrodes as a Function of Potential: The Potential of Zero Charge of Pt(111). *Surf. Sci.* **2004**, *572* (1), 11–22. https://doi.org/10.1016/j.susc.2004.08.014.

(16) Boronat-González, A.; Herrero, E.; Feliu, J. M. Determination of the Potential of Zero Charge of Pt/CO Electrodes Using an Impinging Jet System. *J. Solid State Electrochem.* **2020**, *24* (11–12), 2871–2881. https://doi.org/10.1007/s10008-020-04654-7.

(17) Endo, K.; Katayama, Y.; Miura, T. A Rotating Disk Electrode Study on the Ammonia Oxidation. *Electrochimica Acta* **2005**, *50* (11). https://doi.org/10.1016/j.electacta.2004.09.024.

(18) Bard, Allen J.; Faulkner, Larry R. *Electrochemical Methods: Fundamentals and Applications*, 2nd ed.; John Wiley & Sons, Inc: New York, 2001.
(19) Petersen, H. A.; Miller, E. N.; Pham, P. H.; Kajal; Katsirubas, J. L.; Koltunski, H. J.; Luca, O. R. On the Temperature Sensitivity of Electrochemical Reaction Thermodynamics. *ACS Phys Chem Au* **2023**, *3* (3), 241–251. https://doi.org/10.1021/acsphyschemau.2c00063.
(20) Haynes, W. M. *CRC Handbook of Chemistry and Physics*, 95th ed.; CRC Press: Boca Raton, FL, 2014.
(21) Sander, R. Compilation of Henry's Law Constants (Version 5.0.0) for Water as Solvent. *Atmos Chem Phys* **2023**, *23* (19), 10901–12440. https://doi.org/10.5194/acp-23-10901-2023.
